# Fungal-Bacterial Networks in the *Populus* Rhizobiome Are Impacted by Soil Properties and Host Genotype

**DOI:** 10.3389/fmicb.2019.00481

**Published:** 2019-03-29

**Authors:** Gregory Bonito, Gian Maria Niccolò Benucci, Khalid Hameed, Deborah Weighill, Piet Jones, Ko-Hsuan Chen, Daniel Jacobson, Christopher Schadt, Rytas Vilgalys

**Affiliations:** ^1^Department of Plant Soil and Microbial Sciences, Michigan State University, East Lansing, MI, United States; ^2^Great Lakes Bioenergy Research Center, East Lansing, MI, United States; ^3^Department of Biology, Duke University, Durham, NC, United States; ^4^Biosciences Division, Oak Ridge National Laboratory, Oak Ridge, TN, United States; ^5^The Bredesen Center for Interdisciplinary Research and Graduate Education, University of Tennessee, Knoxville, Knoxville, TN, United States; ^6^Department of Soil and Water Sciences, University of Florida, Quincy, FL, United States

**Keywords:** *Populus deltoides*, fungal communities, bacterial communities, microbiome, NGS sequencing, arbuscular mycorrhizal fungi, endophytes, rhizosphere

## Abstract

Plant root-associated microbial symbionts comprise the plant rhizobiome. These microbes function in provisioning nutrients and water to their hosts, impacting plant health and disease. The plant microbiome is shaped by plant species, plant genotype, soil and environmental conditions, but the contributions of these variables are hard to disentangle from each other in natural systems. We used bioassay common garden experiments to decouple plant genotype and soil property impacts on fungal and bacterial community structure in the *Populus* rhizobiome. High throughput amplification and sequencing of 16S, ITS, 28S and 18S rDNA was accomplished through 454 pyrosequencing. Co-association patterns of fungal and bacterial taxa were assessed with 16S and ITS datasets. Community bipartite fungal-bacterial networks and PERMANOVA results attribute significant difference in fungal or bacterial communities to soil origin, soil chemical properties and plant genotype. Indicator species analysis identified a common set of root bacteria as well as endophytic and ectomycorrhizal fungi associated with *Populus* in different soils. However, no single taxon, or consortium of microbes, was indicative of a particular *Populus* genotype. Fungal-bacterial networks were over-represented in arbuscular mycorrhizal, endophytic, and ectomycorrhizal fungi, as well as bacteria belonging to the orders Rhizobiales, Chitinophagales, Cytophagales, and Burkholderiales. These results demonstrate the importance of soil and plant genotype on fungal-bacterial networks in the belowground plant microbiome.

## Introduction

Living plants, soils and microbiota interact and function in a zone known as the root microbiome (Lundberg et al., 2012). This zone of biological activity is characterized by elevated rates of respiration, nutrient turnover, and carbon sequestration, highlighting its importance to the functioning of terrestrial ecosystems. Root microbiomes continue to be characterized for model and non-model plant species. While rooting, growth, and disease susceptibility are under strong genetic controls in plants (Jorge et al., 2005; Zalesny et al., 2005; Raj et al., 2011), discerning the contribution of host genotype relevant to other ecologically factors in a community context remains a formidable challenge (Lundberg et al., 2012; Peiffer et al., 2013; Shakya et al., 2013; Fonseca-García et al., 2016; Cregger et al., 2018; Fitzpatrick et al., 2018). Here we report on common garden “trap-plant” experiments aimed at disentangling effects of host genotype and soil origin on networks of fungal and bacterial communities that comprise the root microbiome of *Populus deltoides.*

*Populus deltoides* (Poplar/Eastern Cottonwood) is a woody plant that grows along rivers and waterways across much of central and eastern North America (Braatne et al., 2006; Gottel et al., 2011). *Populus deltoides* is particularly well adapted to riparian habitats. This tree species functions in a pioneer and late-stage niche along river banks, establishing from seed or from branches that fall, root and become partially buried during flooding events (Shakya et al., 2013). The disruption of natural flooding cycles may limit the ability of *P. deltoides* to naturally establish (Kranjcec et al., 1998); however, once established *P. deltoides* may persist for centuries. Recruitment processes for *P. deltoides* root-microbiome associates, and their stability and change over time and space, are still not well understood.

Recent efforts have been made to characterize the root microbiome of *Populus* using high throughput sequencing (Gottel et al., 2011; Danielsen et al., 2012; Shakya et al., 2013; Cregger et al., 2018). Gottel et al. (2011) and Shakya et al. (2013) used multiple rDNA regions to target fungal and bacterial rhizosphere and endosphere communities from adult *P. deltoides* trees in their natural habitats. Distinct habitat, seasonal, and geographic differences in the endosphere and rhizosphere communities of *P. deltoides* were found, particularly among bacterial communities. Similar results were reported for pine, agave and *Arabidopsis* (Lundberg et al., 2012; Talbot et al., 2014; Fonseca-García et al., 2016). Host species has also been found to impact the structure of fungi and bacteria within the plant rhizobiome, indicating plant genetic effects (Bonito et al., 2014). The impact of plant genotype on its microbiome appears to be much more important in aboveground tissues (e.g., leaves, stems), compared to in belowground root tissues (Bálint et al., 2013; Cregger et al., 2018). However, differentiating the effects of soil properties from those of host plant genotype still has not been sufficiently addressed.

In this study, two experiments were conducted. The first experiment assessed differences in fungal and bacterial community structure and networks among multiple *Populus* genotypes grown in common soils. The second experiment assessed the influence of fungal and bacterial community structure in the absence of host genetic factors, by growing a single *P. deltoides* genotype in multiple soils in a common environment. In this work, we define the rhizobiome to include fungi and bacteria intimately associated with root tissues, including the rhizoplane and root endosphere, but excluding soil particles associated with the rhizosphere. We used these data to compare levels of α-diversity within genotypes, β-diversity between *Populus* genotypes, and β-diversity levels between a single *Populus* genotype grown in soils originating from different locales. We assessed the fungal-bacterial network structure of root-associated fungal-bacterial communities to determine OTU co-occurance patterns. This research provides a foundation for understanding multi-Kingdom microbial interactions within plant rhizobiomes.

## Materials and Methods

### Trap-Plant Bioassays

Trap-plant bioassay experiments were designed to discern effects of host genotype and soil origin on root microbiomes of *Populus.* In these assays, plant roots serve as “bait” for compatible microbial communities in test soils. Microbes compatible with the plant host persist and grow during the experiment to the point of detection through environmental DNA sequencing, as previously described (Bonito et al., 2014). Field soils from natural *P. deltoides* riparian habitats were collected along the Caney Fork River in TN and the Yadkin River in NC, sites previously sampled by Shakya et al. (2013). Upon collection, fresh soils were refrigerated and used within 5 days by diluting 50% with washed and sterilized fine quartz sand, and homogenized by mixing.

### Experiment 1: Effect of *Populus* Genotype on the Root Microbiome

To examine effects of host genotype on the *Populus deltoides* rhizobiome we used four genotypes that were available: *P. deltoides* D110, D117, D133, and *P. deltoides* × *P. trichocarpa* hybrid 93-968. Winter-harvested cuttings were stored at 4°C for 2 months; 3–4 internode segments of each cutting were rooted by placing in sterile water for 4 weeks under 12 h light. After primary leaves emerged, rooted cuttings were transplanted in triplicate into D40 Deepot cone-tainer Deepots (Stuewe & Sons, Inc.) containing soils collected under *P. deltoides* sites (NC1 and NC2) along the Yadkin River in North Carolina (Shakya et al., 2013). Uninoculated control cuttings (genotypes D110, D112, D113) were planted in sterilized sand alongside experimental treatments. All plants were grown under uniform conditions for the duration of the experiment.

### Experiment 2: Effect of Soil Origin on the Root Microbiome of a Single *Populus* Genotype

A single *Populus* genotype (D124) was planted into 8 soils (TNPo1, TNPo3, TNPo4, TNPo5, TNPo6, TNPo7 TNPo8, TNPo11) collected from along the Caney Fork River in Tennessee (TN), United States, to remove host genotype effects on the development of root-associated microbial communities (Supplementary Table S1). This genotype was chosen because it roots well, and we had a sufficient number of cuttings available for the experimental design. Four replicate cuttings were planted into each of the eight soils, totaling 32 plants. Plants were grown in uniform conditions for the duration of the experiment.

### Soil Collection

An auger core was used to collect samples of approximately 10 L of soil to a depth of 15 cm from each of the ten field sites. Soils were air-dried in paper bags for 1 week and sieved through a 2-mm mesh to homogenize and remove roots. Physical and chemical soil characteristics were determined by the University of Georgia’s Agricultural and Environmental Services Laboratory (AESL)^1^ on composited soil samples from three cores at each site (presented in Supplementary Table S1).

Before planting, soils were mixed to a concentration of 50% by volume into fine textured quartz sand to facilitate water drainage. Incorporated sand had been washed and autoclaved twice over a 2-day period prior to mixing with field soil. Cleanly raised plants were planted into mixed soils using cone-tainer “D-pots” (Steuwe and Sons, Corvallis, OR, United States), which were hung in racks to prevent microbial spread between pots via water. A layer of sterile sand (1-cm) was spread across the surface of each planting to prevent aerial cross-contamination of soils between pots.

### Planting Seedings and Cuttings

*Populus* cuttings (listed above) were stored at 4^°^C in double ziplock bags until use. Before rooting, cuttings were washed in 5% Tween 20 solution to remove adhering particles, the surface was sterilized by submerging in 6% H_2_O_2_ for 10 min and then triple rinsed in tap water. The bottom half of each cutting was soaked in tap water for 24 h in darkness to initiate rooting before planting.

### Growth Conditions, Measurements and Sample Preparations

Plants were grown in the Duke Phytotron under uniform conditions of 18 h days with light levels of 400 μmol/m^2^/s and 6 h nights. Day temperatures were 21.1°C, with humidity of 60%; night temperatures were 15.6°C with 85% humidity. Plants were watered daily and fertilized biweekly with half-strength Hoagland solution. Plants were removed from the growth chamber every month to remove fallen leaf material. At these times, racks were reordered to reduce block effects. Plants were grown for 118 days (Experiment 1) and 165 days (Experiment 2). After this period, plant height was measured as the sum of their shoot length(s) measured from the tip of the apical bud to where it emerged from the rooted cutting.

*Populus* roots were harvested using a retractable razor blade to cut and remove a vertical section of approximately half of the root system. The razor was rinsed in ethanol and flamed between samples to minimize cross-contamination. Soil particles associated with the rhizosphere were removed by placing sampled roots in ziplock bags and washing multiple times with Tween 20. A fine spray of tap water was used to remove any additional adhered soil particles.

### DNA Extractions and Fungal and Bacterial PCR Amplification

Harvested root samples were freeze-dried and ground to a powder in a 2 ml Eppendorf tube using a bead beater and 3 large steel beads. DNA was extracted from ∼500 mg of pulverized root tissue from each plant using the CTAB-based chloroform-isoamyl alcohol (24:1) extraction method (Gardes and Bruns, 1993). To help improve DNA quality, a second sodium acetate ethanol precipitation was done. DNA pellets were eluted in 100 μl of TE buffer. Dilutions of DNA made at 1:3 were used for PCR.

We performed 454 sequencing of amplicon libraries with FLX titanium chemistry and a Roche genome sequencer (Indianapolis, IN, United States). For the genotype experiment, four loci were amplified from each sample: 16S rDNA (V4 region) to target bacteria utilizing primers designed to exclude plastid and mitochondrial organelles (Hodkinson et al., 2012); ITS rDNA to target fungi; 28S rDNA as a second fungal marker, and 18S rDNA to target Glomeromycotina (i.e., arbuscular mycorrhiza taxa) with taxon-specific primers (Lee et al., 2008). A reverse primer (AMrev1_GB) was designed to be specific to AMF and to shorten the amplicon length to be more compatible with 454 sequencing (Bonito et al., 2014). Primer pairs and their sequences were modified to contain the 454 A and B primer, with one of 96 10-bp DNA barcodes on the A primer (see Supplementary Table S2 for complete primer sequence information). The 18S region was not sequenced for the soil origin experiment (Experiment 2).

PCR was carried out in 25 μl reactions using High Fidelity PCR buffer (Invitrogen, Carlsbad, CA, United States), 0.2 mM (each) deoxynucleoside triphosphates (dNTPs), 2 mM MgCl_2_, 0.6 mM forward and reverse primers, 2.5 mg/ml bovine serum albumin (BSA) and 1 unit of Platinum *Taq* (Invitrogen, Carlsbad, CA, United States). To each 25 μl reaction mixture, 1 μl of template DNA (diluted 1:3 in 1x Tris buffer) was added. Thermocycler settings were 5 min at 95°C, then 30 cycles of 95°C for 1 min, 52–62°C for 45 s (annealing temperature differed for each primer pair) and 72°C for 1 min, with a final extension for 7 min at 72°C. Annealing temperatures were optimized with gradient PCR for each primer pair to maximize target amplification and minimize primer dimer. Annealing temperatures were as follows: 58°C for ITS, 62°C for 28S, 52°C for 16S and 55°C for 18S. PCR products were visualized through gel electrophoresis. PCR products from each experiment were then normalized separately by locus (ITS, 28S, 18S, or 16S) to achieve equal molar concentrations of target PCR product for each sample. Pooled PCR products were normalized by molarity into a single library for each experiment such that the library for Experiment 1 included ITS, 16S, 18S, and 28S amplicons, and the library for experiment two included ITS, 16S, and 28S amplicons. Unincorporated primers, dNTPs and primer dimers were removed through two successive rounds with the Agencourt AMPure purification system (Beckman Coulter, Danvers, MA, United States). Product purity and concentration were checked with an Agilent 2100 Bioanalyzer (Santa Clara, CA, United States). Emulsion reactions were performed in paired samples containing two sample PCR amplicons that were matched for template quantity and quality. The two prepared libraries were each loaded into 1/8 of a 454 plate and sequencing was performed on the GS-FLX with Titanium series reagents (Roche), sequencing from the ‘A’ adaptor only (Lib-L) according to manufacturer’s recommended conditions. Sequence data generated during this study have been submitted to GenBank’s sequence read archive under the study accession number SRP106691.

### Assessments of Arbuscular Mycorrhizal Fungal Diversity

Arbuscular mycorrhizal fungi (AMF) are an important and distinct function guild in the *Populus* rhizobiome (Lodge, 1989). To propagate and assess the diversity of *Populus* associated AMF, bioassay plant roots and spores extracted from them were propagated onto sorghum, a host that has been extensively used for maintaining AMF isolates (Selvakumar et al., 2016). After 5 months of growth, sorghum roots were harvested and AMF spores were extracted, sorted morphologically and assessed phylogenetically via 18S rDNA sequencing and analysis (described in Supplementary File S1).

### Bioinformatic Analyses

Raw reads (i.e., *sff*, standard flowgram format) were demultiplexed, cleaned of short and low-quality reads with the spilt_libraries.py script in *QIIME* v1.9 (Caporaso et al., 2010). Default parameters were used with these exceptions: homopolymers were not allowed to exceed 10 bases in length and sequences < 150 and > 1000 bp in length were excluded. Read denoising was done with *ampliconnoise* (Quince et al., 2011) and primer sequences were removed with *cutadapt* (Martin, 2011). For 16S reads, the most hypervariable region was extracted using *metaxa* (Bengtsson et al., 2011). All the reads were then filtered, trimmed and clustered into OTUs at 97% similarity (unless otherwise noted) using the UPARSE pipeline in USEARCH (Edgar, 2010, 2013). OTUs representative sequences of ITS and 16S rDNA were then identified taxonomically inside *QIIME* (with 0.8 bootstrap confidence) with *parallel_assign_taxonomy_rdp.py* script that uses the naïve Bayesian RDP classifier (Wang et al., 2007) against the UNITE sequence reference database (Kõljalg et al., 2013).

We chose to sequence fungal 28S rDNA (LSU) because this region (a) can be aligned across the Kingdom Fungi, allowing for the phylogenetic placement of unknown/understudied taxa, (b) amplifies and sequences easily, and (c) includes variable domains informative at the genus (and sometimes species) level for identification of taxa. 28S rDNA sequences were processed with QIIME (Caporaso et al., 2010). Chimeric sequences were removed with USEARCH (Edgar, 2010). Sequences were mapped according to the eukaryotic SILVA LSU reference database that was parsed locally to include fungal and non-fungal representative sequences. Sequences mapping to non-fungal taxa were filtered from the dataset. OTUs were assigned by clustering the sequences at 99% similarity, as recommended by Bonito et al. (2014). Taxonomy was assigned to representative sequences with a 28S rDNA reference sequences database compiled from the curated RDP dataset. Taxonomies of abundant taxa were verified by comparing sequences to the NCBI database with the BLAST algorithm. Neighbor joining trees were built using PAUP 4.0 (Swofford, 2003). Trees were analyzed using the unweighted UniFrac metric calculated with Fast UniFrac (Hamady et al., 2009) and samples were categorized according to sample host genotype and soil origin. The UniFrac significance test allows one to examine differences among treatments based solely on branch length data while the P-test is based on a phylogenetic tree to test whether two environments are significantly different by using parsimony-based scoring significances (Lozupone and Knight, 2005). Both of these tests were used to assess for phylogenetic differences in the fungal communities associated with different host genotypes and soils based on 1,000 permutations and calculated with the Fast UniFrac as implemented in QIIME.

We sequenced 18S rDNA (SSU) with the aim of selectively detecting Glomeromycotina (AMF). Chimeric sequences were first removed with USEARCH (Edgar, 2010). The sequences were then mapped to a version of the eukaryotic SILVA SSU reference database, which includes fungal and non-fungal representative sequences. Sequences not mapping to Glomeromycotina from the dataset were excluded. Remaining OTUs were assigned by clustering the sequences at 98% similarity following Bonito et al. (2014). To ascertain the phylogenetic placement of these OTUs a representative sequence from each OTU was aligned with sequences belonging to reference taxa from across the Glomeromycotina (Kruger et al., 2012) with the alignment program MUSCLE (Edgar, 2004). Phylogenetic analyses were carried out in PAUP 4.0 (Swofford, 2003) as previously described.

### Statistical Analysis

Rarefaction curves were assessed for each sample (Supplementary Figure S1) and OTU table values were normalized though Hellinger transformation (Legendre and Gallagher, 2001; Pezzolla et al., 2015). Principal coordinates analysis (PCoA) was used to represent inter-sample Bray Curtis dissimilarity. The ordinate function of the R (R Core Team, 2018) package “phyloseq” (McMurdie and Holmes, 2013) was used to produce graphs. Permutational multivariate analysis of variance (PERMANOVA; Anderson, 2001) was used to test the null hypothesis of no differences among *a priori* defined groups with the function adonis in the “vegan” R package (Oksanen et al., 2015) with 9999 permutations. Permutational analysis of multivariate dispersions (Anderson, 2006) was used to test for variance heterogeneity between the *a priori* selected groups using the function betadisper in the “vegan” R package. A taxon-group association analysis was used to assess the degree of preference (at *P* ≤ 0.05, after FDR correction) of each OTU for the target group in relation to other groups using the multipath function of the “indicspecies” R package (De Cáceres et al., 2010). Bar plots of relative abundance taxonomic distribution were calculated using the R package RAM (Chen et al., 2016).

### Experiment 1: Effect of *Populus* Genotype on the Root Microbiome

OTU networks have been used in the study of plant and microbial communities (Maestre et al., 2009; Faust and Raes, 2016). We constructed microbial OTU networks in order to investigate the effect of the *Populus* genotype on the root microbiome. Data were represented in the form of a Hellinger-transformed OTU matrix in which rows represented OTUs and columns represented samples. Networks were visualized in Cytoscape (Shannon et al., 2003) (Supplementary File S2).

### Raw Bipartite and Sample Correlation Networks

Bipartite networks were constructed from the OTU matrix by representing OTUs and samples as nodes and connecting an OTU node to a sample node if the OTU was present in that sample. Sample similarity networks were constructed by calculating the similarity between all pairs of columns (samples) of the OTU matrix and then constructing a Maximum Spanning Tree (MST). Two similarity metrics were used, the Pearson correlation coefficient and Proportional Similarity (Bloom, 1981; Weighill and Jacobson, 2017). Pearson correlation coefficients were calculated using the mcxarray and mcxdump programs from the MCL-Edge software package (Van Dongen, 2008) available from http://micans.org/mcl/. MST construction was performed by converting Pearson correlations to distance measures by taking (1 – pearson) and then making use of the Dijkstra minimum spanning tree algorithm in the Graph Perl module (Jarkko Hietaniemi)^2^. Resulting MSTs were visualized as networks in Cytoscape (Shannon et al., 2003) and sample nodes were colored according to genotype and soil.

### OTU-Sample Enrichment Networks (Hypergraphs)

OTU-Sample enrichment networks (hypergraphs) were constructed using the Fisher Exact Test, similar to the enrichment networks/hypergraphs constructed previously (Weighill and Jacobson, 2015). The samples for Experiment 1 were separated into control samples and non-control samples (including NC1 and NC2), resulting in a matrix M in which rows represented OTUs and columns represented non-control samples. For each non-control sample and for each OTU, the contingency table (Supplementary Figure S2) was constructed and the Fisher Exact test was performed on the contingency table. The Text::NSP::Measures::2D::Fisher Perl module (Banerjee and Pedersen, 2003), available from Comprehensive Perl Archive Network (CPAN) at http://search.cpan.org/dist/Text- xNSP/lib/Text/NSP/Measures/2D/Fisher.pm, was used to calculate the Fisher Exact Test. False discovery rate correction of *p*-values (Benjamini and Hochberg, 1995) at a level of 0.01 determined which OTUs were enriched in which samples. An enrichment network was constructed by connecting a sample node to an OTU node if that OTU was significantly enriched in that sample. Similarly, enrichment of OTUs in control samples was performed.

### Binned Enrichment Networks

We constructed binned enrichment networks to determine which OTUs were enriched in which genotypes, separately within each soil. Within each soil (i.e., Control, NC1, and NC2) the data were binned by genotype. This resulted in a separate matrix for each soil in which rows represented OTUs, columns represented genotypes and each entry represented the collective amount of that OTU present in that genotype in that particular soil. Enrichment and FDR correction were performed separately for each soil. For each OTU and each genotype within a particular soil, the Fisher Exact test was calculated similarly as described above, and FDR correction at a level of 0.01 was performed. This was repeated for each soil. An enrichment network was then constructed representing the enrichment of OTUs in genotypes within each soil.

### Community Construction

Communities of OTUs were constructed as follows: OTUs with less than 3 non-zero values were removed, and the Pearson correlation between all pairs of remaining OTUs was calculated across all samples of experiment 1 focused on plant genotype impacts on the plant microbiome. This made use of the ‘mcxarray’ and ‘mcxdump’ programs from the MCL-Edge software package (Van Dongen, 2008) available from http://micans.org/mcl/. An absolute threshold of 0.6 was set, and numerous topology measures were calculated for the correlation network with the NetworkAnalyzer Cytoscape app (Assenov et al., 2008). Communities were constructed by clustering the network with the Markov Cluster (MCL) algorithm (Van Dongen, 2008), with an inflation value of 2.5 using the cluster Maker Cytoscape app (Morris et al., 2011) (Supplementary File S3).

### Differential Analysis

The differential relative abundance was estimated using the Fold Change Rank Order Statistic (FCROS) package in R (Dembélé and Kastner, 2014). To control for the bias of rare OTUs we required that an OTU’s abundance measurement be present in at least 3 of the samples. A variation stabilization transformation, as suggested in McMurdie and Holmes (2014), was applied by performing a log transform. A quantile-normalization procedure (Amaratunga and Cabrera, 2001) was applied to transformed abundance values to account for noise between sample replicates, while maintaining variation between sample classes. The normalized matrix of values was analyzed using FCROS for differential relative abundance of OTUs between sample classes. A *p*-value threshold of 0.05 was applied and using *f*-value output of FCROS, OTUs with a probability of over/under abundance greater than 0.9, respectively, was maintained for further analysis.

### Experiment 2: Effect of Soil Origin on the Root Microbiome of a Single *Populus* Genotype

A similar series of analyses were performed for Experiment 2 as described for Experiment 1. Data were first summarized in the form of a Hellinger-transformed OTU matrix in which rows represented OTUs and columns represented samples of *Populus* roots grown in different soil samples.

### Sample Similarity and OTU-Sample Enrichment Networks (Hypergraphs)

Sample similarity MSTs were constructed for the samples in Experiment 2 using the Pearson correlation coefficient and proportional similarity, in a similar manner as described for Experiment 1. The resulting MSTs were represented as networks in Cytoscape (Shannon et al., 2003) and nodes were colored based on various properties. OTU-sample enrichment networks representing the enrichment of OTUs in samples were constructed similarly as described in Experiment 1, making use of the Fisher Exact test and false discovery rate correction.

### Community Construction

OTU communities were constructed for Experiment 2 in a similar manner as described for Experiment 1, by removing OTUs with fewer than 3 non-zero values across samples, calculating the Pearson correlation between all pairs of OTUs across all Experiment 2 samples, applying an absolute threshold of 0.6 and clustering with MCL. Network topology measures were again calculated using the NetworkAnalyzer Cytoscape app (Morris et al., 2011).

### Soil Property Correlation

A soil property similarity network was constructed by calculating the Pearson correlation coefficient between all pairs of soil properties across all soils in Experiment 2. This made use of the mcxarray and mcxdump programs from the MCL-Edge software package (Van Dongen, 2008) available from http://micans.org/mcl/. A maximum spanning tree was constructed from the resulting network using the Dijkstra algorithm in the Graph Perl module (Jarkko Hietaniemi)^3^. In addition, the Pearson correlation between all soil properties and OTUs was calculated. After applying a threshold of |Pearson|≥ 0.6, soil properties correlating with OTUs were mapped onto the corresponding communities.

### Differential Analysis

Significant differences in the root microbiome under the effect of different soils were assessed through differential analysis, similar to Experiment 1. Filtering out rare OTUs that only occur in fewer than 3 samples, a variance stabilization transformation and quantile normalization were performed to mitigate noise between sample replicates. Relative abundances of OTUs in the respective sample classes, different soils, were then compared using FCROS. A *p*-value threshold of 0.05 and *f*-value threshold of 0.9 were applied.

### Plant Growth Analyses

Effects of eight different soils on the growth of a single cottonwood genotype (D124) were assessed with a one-way ANOVA using R version 2.15.2. Normality was checked graphically with normal quantile–quantile plots and computationally with the Shapiro–Wilk test of normality. Homoscedasticity was assessed by calculating the variances of the data grouped by the levels of each factor and comparing the values to see if any were more than twice any of the others. Since this was often the case, a Bartlett test of homoscedasticity was used, which evaluates the null hypothesis of equal variances. Differences between means in ANOVAs were checked *a posteriori* with the Tukey HSD test. Growth data were right-skewed slightly, so they were log-transformed to meet the assumptions of normality and homoscedasticity. Chi-square tests were used to check whether differences in OTUs detected between different host genotypes and soils were significant.

## Results

### PCR and 454 Sequencing

Sixty-four plants were grown and harvested for the two experiments described above. Two 454 libraries were prepared with amplicon pools from multiplexed samples and were successfully sequenced. The first library was prepared for the host genotype experiment and included amplicon pools of ITS, 18S, 28S and 16S rDNA from 34 samples and resulted in 113,869 reads. After error-correction and filtering for quality, length and chimeras 14,847 sequences of ITS, 16,671 of 18S (374 of which mapped to Glomeromycotina), 44,817 sequences of 28S and 9,822 sequences of 16S were recovered. The 34 samples had between 68 and 1950 reads per sample per locus (mean = 643). The second library was prepared for the soil origin experiment and included amplicon pools of ITS, 28S, and 16S rDNA from 30 samples and resulted in 129,674 reads. After error-correction and filtering for quality, length and chimeras, 44,938 sequences of ITS, 44,155 of 28S and 21,948 sequences of 16S were recovered. One sample failed to amplify and/or sequence well and was excluded from further analyses. The remaining 29 samples had between 346 and 4065 reads per sample per locus (mean = 1276).

### Experiment 1: Effect of *Populus* Genotype on the Root Microbiome

**FIGURE 1:**
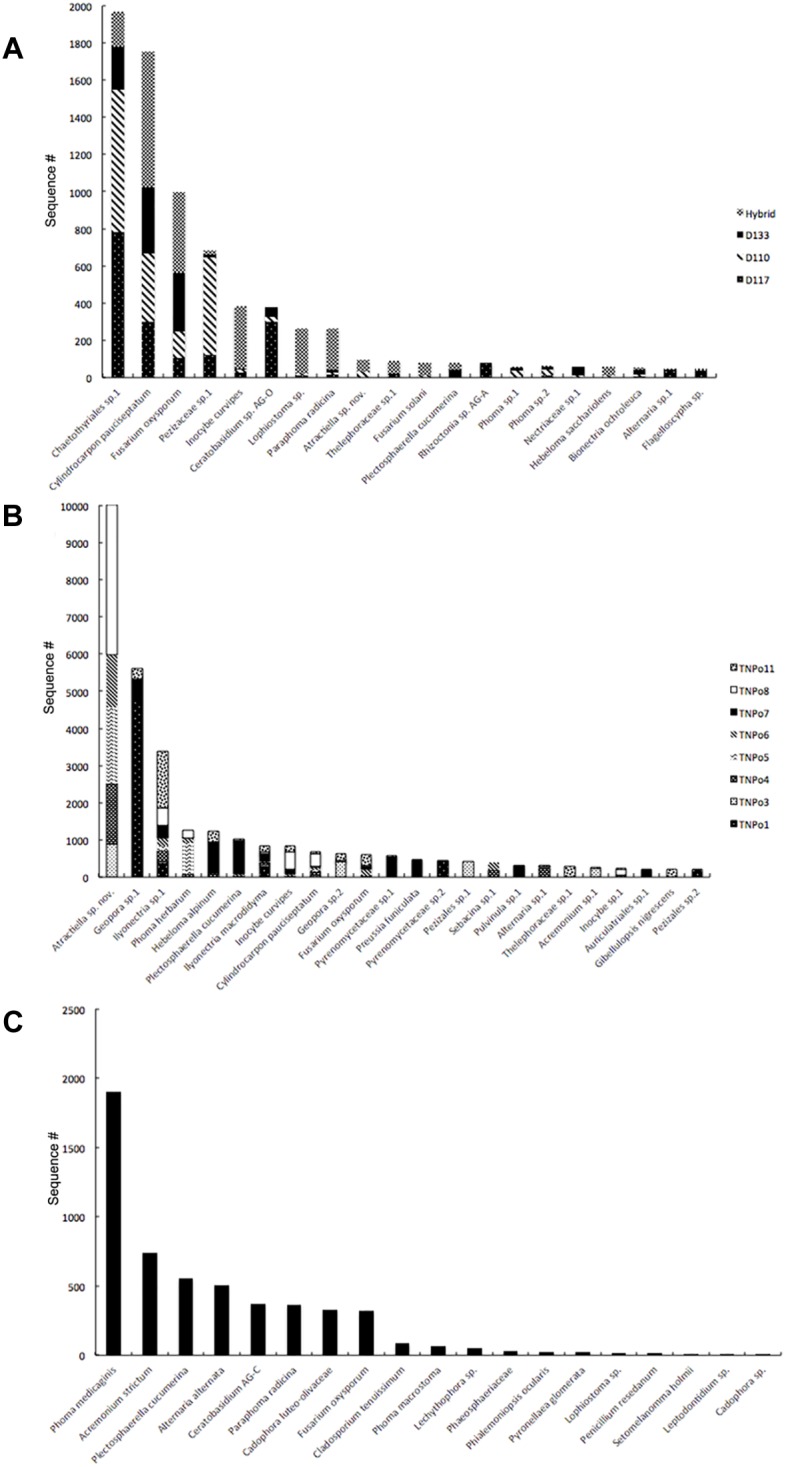
Rank abundance of fungi in *Populus* roots based on ITS rDNA amplicon sequencing. **(A)** Across different genotypes (Experiment 1); **(B)** Across different soils (Experiment 2); **(C)** In uninoculated control plants.

**FIGURE 2:**
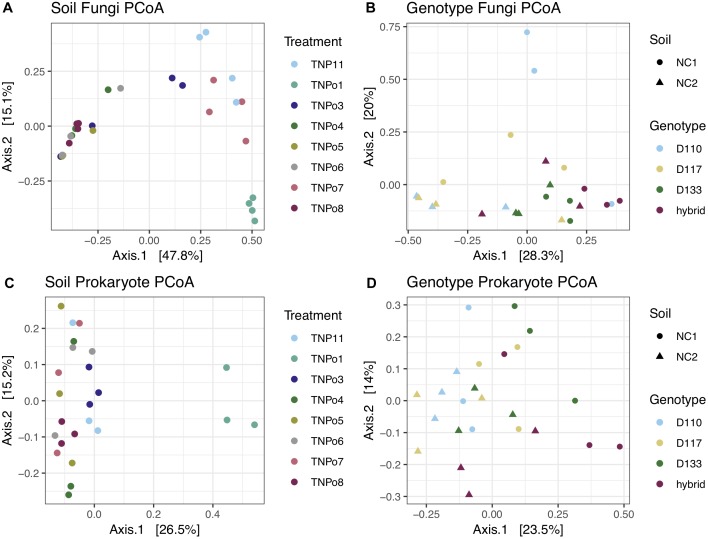
Principle coordinates analysis (PCoA) showing beta-diversity of fungal and prokaryotic communities in the rhizosphere of *Populus*. **(A)** Fungal communities in different soils (Experiment 2); **(B)** Fungal communities in different *Populus* genotypes (Experiment 1); **(C)** Prokaryotic communities in different soils (Experiment 2); **(D)** Prokaryotic communities in different *Populus* genotypes (Experiment 1).

Significant differences in fungal root-associated communities could be attributed to host genotype, but were not associated with specific OTUs. The most abundant fungal OTUs (based on ITS1) belonged to Chaetothyriales sp.1, *Cylindrocarpon pauciseptatum* ( = *Ilyonectria*), *Fusarium oxysporum*, Pezizaceae sp. 1 and *Inocybe curvipes* and were detected on all genotypes assayed in this experiment (Figure 1A). ITS1 data revealed 161 OTUs in this dataset. Only 5–8 unique OTUs were detected on any particular genotype, and 99% of these were rare—that is detected < 10 times and on fewer than half of the replicates (Supplementary Table S3). PCoA of ITS and 28S rDNA showed that samples were separated by soil, rather than by genotype, in ordinal space (Figure 2A,B). No significant differences in OTU richness or UniFrac distances based on 28S were realized between the tested genotypes. PERMANOVA of the fungal ITS (*R*^2^ = 0.13, *p* = 0.157, perm = 9999) and 16S (*R*^2^ = 0.13, *p* = 0.10, perm = 9999) data showed no significant interactions between soil and plant genotype. Therefore, genotype effects were tested separately on the two soils used in this experiment. For the NC1 soil, significant effects of plant genotype were found for fungal (*R*^2^ = 0.40, *p* = 0.040, perm = 9999) and bacterial communities (*R*^2^ = 0.37, *p* = 0.038, perm = 9999). Multivariate dispersion (variances) of sample groups did not differ statistically for fungi (*F* = 0.245, *p* = 0.868) or bacteria (*F* = 0.565, *p* = 0.625), respectively, validating results of PERMANOVA. However, for the NC2 soil no significant genotype effects were found for either fungal (*R*^2^ = 0.252, *p* = 0.140, perm = 9999) or bacterial communities (*R*^2^ = 0.365, *p* = 0.166, perm = 9999). Significant effects of soil origin were found for both fungal (*R*^2^ = 0.123, *p* = 0.0008, perm = 9999) and bacterial communities (*R*^2^ = 0.134, *p* = 0.0001, perm = 9999). No difference in group dispersion was detected for fungal (*F* = 1.30, *p* = 0.269) or bacterial (*F* = 0.497, *p* = 0.493) communities. All *Populus* genotypes tested harbored a diverse community of root-associated bacteria dominated by *Niastella*, Micromonosporineae, and *Bradyrhizobium* (Supplementary Figure S3).

Fungal and bacterial communities of the root microbiome between treatment and control plants grown without soil inoculum differed drastically. Control plants largely clustered together in PCoA (Figure 2B,C). In general, uninoculated control plants were colonized by a lower diversity of bacteria than plants grown in soil. *Flavobacterium*, Streptomycineae, Rhizobiaceae, and *Stenotrophomonas* were most abundant on control plants. Eight fungal species belonging to the genera *Phoma, Acremonium, Plectosphaerella, Alternaria, Ceratobasidium, Paraphoma, Cadophora*, and *Fusarium* were most abundant in the control plants, independent of plant genotype (Figure 1C). Of these, only *Fusarium oxysporum* and *Paraphoma radicina* were detected at modest levels in both control and treatment plants. Bacterial communities in the controls were all varied across genotype but different between cuttings planted in soil (Figure 2).

### Indicator Species Analysis

No indicator species of fungi or bacteria were found to be associated to any specific genotype (Table 1). However, two dominant fungal OTUs, Chaetothyriales sp., and *Cylindrocarpon pauciseptatum*, were significantly associated with the two NC soils used in Experiment 1. OTUs belonging to *Polyscytalum pustulans*, Pleosporales sp., *Sarocladium strictum, Cladosporium ramotenellum*, *Lecythophora fasciculata*, and *Cadophora luteo-olivacea* were found to be significantly associated with uninoculated control plants. Likewise, numerous bacterial OTUs were associated with control plants (Table 1). Conversely, bacteria belonging to Micromonosporaceae, Chitinophagaceae, and Oxalobacteraceae were significantly associated with experimental soils, but not uninoculated control plants.

**Table 1:** Indicator species analysis of fungal and bacteria operational taxonomic units (OTUs) that are statistically associated with a particular *Populus* genotype.

Soil fungi	OTU	Treatment	*p*-value after FDR									
	OTU_52	TNP11	0.00891	^∗∗^	OTU_52	Fungi	Basidiomycota	Agaricomycetes	Thelephorales	Thelephoraceae		
	OTU_40	TNPo1	0.026	^∗^	OTU_40	Fungi	Ascomycota	Pezizomycetes	Pezizales	Pyronemataceae	Unidentified	Pyronemataceae sp
	OTU_118	TNPo3	0.0078	^∗∗^	OTU_118	Fungi	Basidiomycota	Agaricomycetes	Agaricales	Niaceae	Flagelloscypha	Flagelloscypha minutissima
	OTU_30	TNPo6	0.0468	^∗^	OTU_30	Fungi	Basidiomycota	Agaricomycetes	Agaricales			
	OTU_51	TNPo6	0.0481	^∗^	OTU_51	FungiPMI	Ascomycota	Sordariomycetes	unclassified	Unclassified	Unclassified	Sordariomycetes_sp_PMI488
	OTU_22	TNPo7	0.0078	^∗∗^	OTU_22	Fungi	Ascomycota	Pezizomycetes	Pezizales	Tuberaceae	Tuber	Tuber mexiusanum
	OTU_16	TNPo7	0.0281	^∗^	OTU_16	Fungi	Ascomycota	Pezizomycetes	Pezizales	Incertae	Sedis	Pulvinula sp
	OTU_4	TNP11+TNPo7	0.0234	^∗^	OTU_4	Fungi	Basidiomycota	Agaricomycetes	Agaricales	Strophariaceae	Hebeloma	Hebeloma cavipes
	OTU_2	TNP11+TNPo1+TNPo7	0.0052	^∗∗^	OTU_2	Fungi	Ascomycota	Pezizomycetes	Pezizales	Pyronemataceae	Geopora	
	OTU_56	TNPo5+TNPo6+TNPo8	0.0078	^∗∗^	OTU_56	FungiPMI	Ascomycota	Sordariomycetes	Magnaporthales	Magnaporthaceae	Mycoleptodiscus	Mycoleptodiscus_sp_PMI219
	OTU_8	TNP11+TNPo6+TNPo7+TNPo8	0.0052	^∗∗^	OTU_8	Fungi	Basidiomycota	Agaricomycetes	Agaricales	Inocybaceae	Inocybe	Inocybe curvipes
	OTU_1	TNPo3+TNPo4+TNPo5+TNPo6+TNPo8	0.0052	^∗∗^	OTU_1	Fungi	Basidiomycota	Atractiellomycetes	Atractiellales	Unidentified	Unidentified	Atractiellales sp
Soil genotype	None											
Soil genotype by soil	OTU_14	Control	0.00144	^∗∗^	OTU_14	Fungi	Ascomycota	Incertae sedis	Incertae sedis	Incertae sedis	Polyscytalum	Polyscytalum pustulans
	OTU_18	Control	0.00144	^∗∗^	OTU_18	Fungi	Ascomycota	Dothideomycetes	Pleosporales	Incertae sedis	Phoma	NA
	OTU_8	Control	0.00144	^∗∗^	OTU_8	Fungi	Ascomycota	Dothideomycetes	Pleosporales	Unclassified	Unclassified	PleosporalePMI117
	OTU_5	Control	0.00144	^∗∗^	OTU_5	Fungi	Ascomycota	Sordariomycetes	Hypocreales	Incertae sedis	Sarocladium	Sarocladium strictum
	OTU_1	Control	0.00144	^∗∗^	OTU_1	Fungi	Ascomycota	Dothideomycetes	Pleosporales	Incertae sedis	Phoma	NA
	OTU_9	Control	0.01313	^∗^	OTU_9	Fungi	Ascomycota	Sordariomycetes	NA			
	OTU_16	Control	0.00253	^∗∗^	OTU_16	Fungi	Ascomycota	Dothideomycetes	Capnodiales	Davidiellaceae	Cladosporium	Cladosporium ramotenellum
	OTU_46	Control	0.00337	^∗∗^	OTU_47	Fungi	Ascomycota	Dothideomycetes	Pleosporales	Unclassified	Unclassified	PleosporalePMI628
	OTU_31	Control	0.02693	^∗^	OTU_31	Fungi	Ascomycota	Sordariomycetes	Coniochaetales	Coniochaetaceae	Lecythophora	Lecythophora fasciculata
	OTU_21	Control	0.0202	^∗^	OTU_21	Fungi	Ascomycota	Leotiomycetes	Helotiales	Incertae_sedis	Cadophora	Cadophora_luteo-olivacea__PMI1442
	OTU_3	NC1+NC2	0.00144	^∗∗^	OTU_3	Fungi	Ascomycota	NA				
	OTU_2	NC1+NC2	0.00144	^∗∗^	OTU_2	Fungi	Ascomycota	Sordariomycetes	Hypocreales			
Bacteria soil	OTU_319	TNP11+TNPo1+TNPo4	0.0171	^∗^	OTU_229	Bacteria	Proteobacteria	Alphaproteobacteria	Rhodobacterales	Hyphomonadaceae		
	OTU_229	TNP11+TNPo4+TNPo5+TNPo6+TNPo8	0.0171	^∗^	OTU_319	Bacteria	Bacteroidetes	[Saprospirae]	[Saprospirales]	Chitinophagaceae		
Bacteria genotype	None											
Bacteria genotype by soil	OTU_36	Control	0.00223	^∗∗^	OTU_36	Bacteria	Proteobacteria	Betaproteobacteria	Burkholderiales	Comamodaceae		
	OTU_46	Control	0.00394	^∗∗^	OTU_46	Bacteria	Proteobacteria	Betaproteobacteria	Burkholderiales	Comamodaceae	Stenotrophomos	
	OTU_41	Control	0.00582	^∗∗^	TU_41	Bacteria	Proteobacteria	Gammaproteobacteria	Xanthomodales	Xanthomodaceae	Janthinobacterium	
	OTU_6	Control	0.01195	^∗^	OTU_6	Bacteria	Proteobacteria	Betaproteobacteria	Burkholderiales	Oxalobacteraceae	Salinispora	
	OTU_21	Control	0.00223	^∗∗^	OTU_21	Bacteria	Proteobacteria	Betaproteobacteria	Burkholderiales	Burkholderiaceae	Flavobacterium	*Flavobacterium succinicans*
	OTU_1	Control	0.00223	^∗∗^	OTU_1	Bacteria	Bacteroidetes	Flavobacteriia	Flavobacteriales	Flavobacteriaceae	Nocardioides	
	OTU_166	Control	0.00394	^∗∗^	OTU_166	Bacteria	Actinobacteria	Actinobacteria	Actinomycetales	Nocardioidaceae	Cytophaga	
	OTU_44	Control	0.00319	^∗∗^	OTU_44	Bacteria	Bacteroidetes	Cytophagia	Cytophagales	Cytophagaceae	Asticcacaulis	
	OTU_52	Control	0.01402	^∗^	OTU_52	Bacteria	Proteobacteria	Alphaproteobacteria	Caulobacterales	Caulobacteraceae		
	OTU_51	Control	0.00803	^∗∗^	OTU_51	Bacteria	Bacteroidetes	Sphingobacteriia	Sphingobacteriales	Sphingobacteriaceae	Prosthecobacter	
	OTU_33	Control	0.00394	^∗∗^	OTU_33	Bacteria	Verrucomicrobia	Verrucomicrobiae	Verrucomicrobiales	Verrucomicrobiaceae	Aminobacter	
	OTU_39	Control	0.00582	^∗∗^	OTU_39	Bacteria	Proteobacteria	Alphaproteobacteria	Rhizobiales	Phyllobacteriaceae	Pseudonocardia	*Pseudonocardia-halophobica*
	OTU_11	Control	0.01223	^∗^	OTU_11	Bacteria	Actinobacteria	Actinobacteria	Actinomycetales	Pseudonocardiaceae		
	OTU_15	Control	0.04733	^∗^	OTU_15	Bacteria	Proteobacteria	Alphaproteobacteria	Ellin329		Sphingobium	
	OTU_180	Control	0.00909	^∗∗^	OTU_180	Bacteria	Proteobacteria	Alphaproteobacteria	Sphingomodales	Sphingomodaceae	Novosphingobium	
	OTU_60	Control	0.04262	^∗^	OTU_60	Bacteria	Proteobacteria	Alphaproteobacteria	Sphingomodales	Sphingomodaceae		
	OTU_212	Control	0.04733	^∗^	OTU_212	Bacteria	Proteobacteria	Gammaproteobacteria	Pseudomodales	Pseudomodaceae	Chryseobacterium	
	OTU_183	Control	0.04555	^∗^	OTU_183	Bacteria	Bacteroidetes	Flavobacteriia	Flavobacteriales	[Weeksellaceae]	Alicyclobacillus	
	OTU_167	Control	0.01402	^∗^	OTU_167	Bacteria	Firmicutes	Bacilli	Bacillales	Alicyclobacillaceae		
	OTU_176	Control	0.02058	^∗^	OTU_176	Bacteria	Verrucomicrobia	[Pedosphaerae]	[Pedosphaerales]	auto67_4W		
	OTU_69	Control	0.01567	^∗^	OTU_69	Bacteria	Proteobacteria	Alphaproteobacteria	Rhodospirillales	Acetobacteraceae		
	OTU_170	Control	0.02973	^∗^	OTU_170	Bacteria	Bacteroidetes	Sphingobacteriia	Sphingobacteriales	Sphingobacteriaceae		
	OTU_7	Control	0.01549	^∗^	OTU_7	Bacteria	Bacteroidetes	[Saprospirae]	[Saprospirales]	Chitinophagaceae		
	OTU_35	Control	0.02883	^∗^	OTU_35	Bacteria	Proteobacteria	Alphaproteobacteria	Rhizobiales			
	OTU_10	Control	0.0363	^∗^	OTU_10	Bacteria	Acidobacteria	Solibacteres	Solibacterales		Chitinophaga	
	OTU_113	NC1	0.00223	^∗∗^	OTU_113	Bacteria	Bacteroidetes	[Saprospirae]	[Saprospirales]	Chitinophagaceae		
	OTU_108	NC1	0.00858	^∗∗^	OTU_108	Bacteria	Armatimodetes	Armatimodia	Armatimodales	Armatimodaceae		
	OTU_270	NC1	0.04555	^∗^	OTU_270	Bacteria	Bacteroidetes	[Saprospirae]	[Saprospirales]	Chitinophagaceae		
	OTU_130	NC2	0.00223	^∗∗^	OTU_130	Bacteria	Actinobacteria	Actinobacteria	Actinomycetales	Micromonosporaceae		
	OTU_159	NC2	0.00469	^∗∗^	OTU_159	Bacteria	Bacteroidetes	[Saprospirae]	[Saprospirales]	Chitinophagaceae		
	OTU_138	NC2	0.00469	^∗∗^	OTU_138	Bacteria	Proteobacteria	Betaproteobacteria	Burkholderiales	Oxalobacteraceae		
	OTU_226	NC2	0.00319	^∗∗^	OTU_226	Bacteria	Bacteroidetes	[Saprospirae]	[Saprospirales]	Chitinophagaceae	Asticcacaulis	
	OTU_142	NC2	0.00582	^∗∗^	OTU_142	Bacteria	Proteobacteria	Alphaproteobacteria	Caulobacterales	Caulobacteraceae		
	OTU_139	NC2	0.01702	^∗^	OTU_139	Bacteria	Chloroflexi	Ktedonobacteria	Ktedonobacterales	Ktedonobacteraceae	Phenylobacterium	
	OTU_145	NC2	0.04207	^∗^	OTU_145	Bacteria	Proteobacteria	Alphaproteobacteria	Caulobacterales	Caulobacteraceae	Caulobacter	
	OTU_17	Control+NC1	0.014	^∗^	OTU_17	Bacteria	Proteobacteria	Alphaproteobacteria	Caulobacterales	Caulobacteraceae		
	OTU_16	Control+NC2	0.00743	^∗∗^	OTU_16	Bacteria	Proteobacteria	Alphaproteobacteria	Rhizobiales	Rhizobiaceae	Asticcacaulis	Asticcacaulis biprosthecium
	OTU_40	Control+NC2	0.02883	^∗^	OTU_40	Bacteria	Proteobacteria	Alphaproteobacteria	Caulobacterales	Caulobacteraceae		
	OTU_76	NC1+NC2	0.00223	^∗∗^	OTU_76	Bacteria	Bacteroidetes	[Saprospirae]	[Saprospirales]	Chitinophagaceae		
	OTU_129	NC1+NC2	0.00223	^∗∗^	OTU_129	Bacteria	Proteobacteria	Betaproteobacteria	Burkholderiales	Comamodaceae		
	OTU_99	NC1+NC2	0.00223	^∗∗^	OTU_99	Bacteria	Actinobacteria	Actinobacteria	Actinomycetales	Micromonosporaceae		
	OTU_93	NC1+NC2	0.00223	^∗∗^	OTU_93	Bacteria	Actinobacteria	Actinobacteria	Actinomycetales	Micromonosporaceae		
	OTU_80	NC1+NC2	0.00319	^∗∗^	OTU_80	Bacteria	Proteobacteria	Gammaproteobacteria	Xanthomodales	Sinobacteraceae	Telmatospirillum	
	OTU_88	NC1+NC2	0.00223	^∗∗^	OTU_88	Bacteria	Proteobacteria	Alphaproteobacteria	Rhodospirillales	Rhodospirillaceae		
	OTU_77	NC1+NC2	0.00319	^∗∗^	OTU_77	Bacteria	Actinobacteria	Actinobacteria	Actinomycetales	Actinospicaceae		
	OTU_79	NC1+NC2	0.00558	^∗∗^	OTU_79	Bacteria	Armatimodetes	Armatimodia	FW68			
	OTU_75	NC1+NC2	0.01223	^∗^	OTU_75	Bacteria	Actinobacteria	Actinobacteria	Actinomycetales	Actinospicaceae		
	OTU_106	NC1+NC2	0.01402	^∗^	OTU_106	Bacteria	Bacteroidetes	[Saprospirae]	[Saprospirales]	Chitinophagaceae	Niastella	
	OTU_112	NC1+NC2	0.01223	^∗^	OTU_112	Bacteria	Bacteroidetes	[Saprospirae]	[Saprospirales]	Chitinophagaceae		


*Significance codes: “^∗^” 0.05, “^∗∗^” 0.01 fater 9999 permutations.*

### Arbuscular Mycorrhizal Communities

**FIGURE 3:**
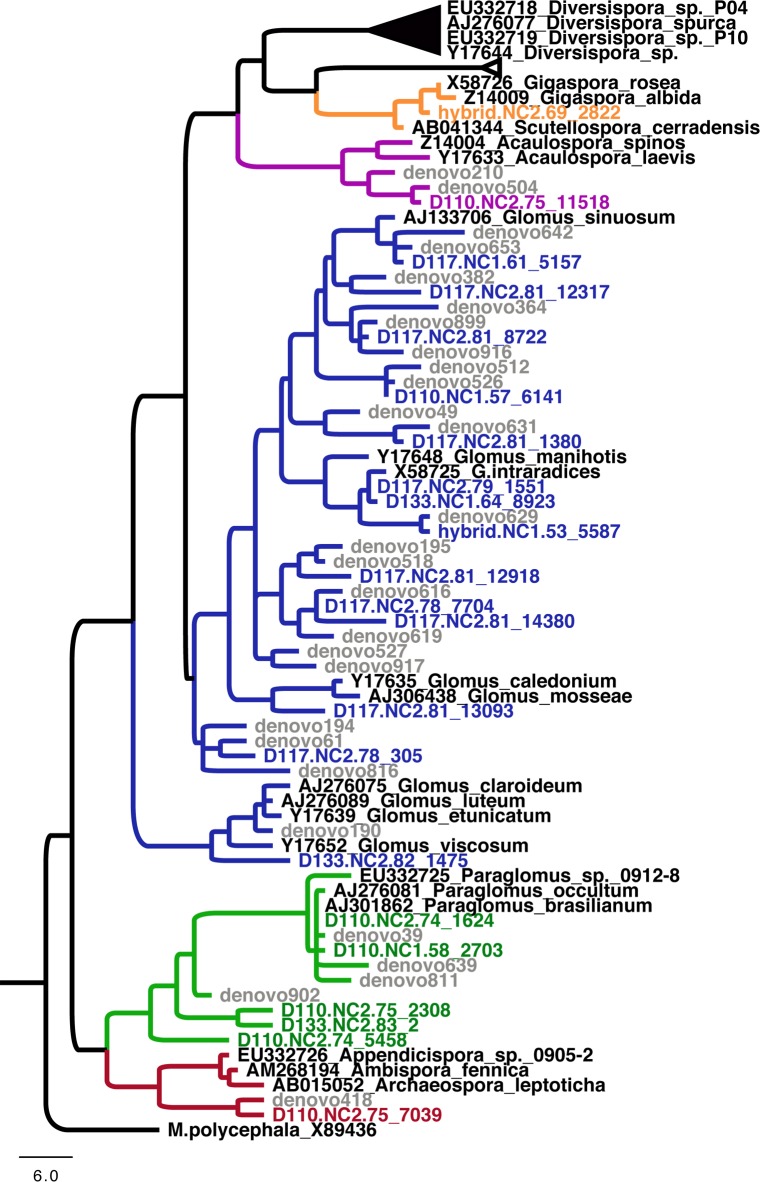
Phylogeny of Glomeromycotina showing the phylogenetic position of arbuscular mycorrhizal fungi detected in this study. Taxonomic families are denoted by colored branches. Black branches denote Diversisporaceae; orange branches denote Gigasporaceae; blue branches denote Glomeraceae; green branches denote Paraglomeraceae; and red branches denote Archaeosporaceae.

In total, 22 OTUs belonging to the Glomeromycotina were detected across these genotypes (Figure 3 and Supplementary File S1); aside from a single OTU belonging to *Gigaspora* detected on the hybrid genotype, no discernable effects of genotype on their distribution were evident. By genotyping arbuscular mycorrhizal spores (60 sequences, 10 OTUs), we detected that sorghum enriches for a different AMF community compared to *Populus* (only 3 overlap OTU between *Populus* root pyrosequencing and Sanger genotyping of sorghum roots). The most prevalent AMF orders detected on *Populus* were Glomerales, Paraglomerales, Diversisporales and Archaeosporales, respectively (Supplementary File S1).

### Plant Genotype Impact on Fungal and Bacterial Networks

**FIGURE 4:**
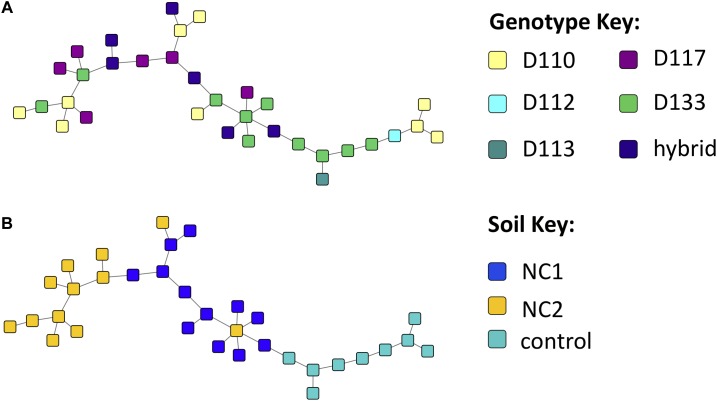
Experiment 1 sample similarity Maximum Spanning Trees (MSTs), constructed using the Proportional Similarity metric, representing similarity of samples based on their Operational taxonomic unit (OTU) content, colored by **(A)** soil and **(B)** genotype.

**FIGURE 5:**
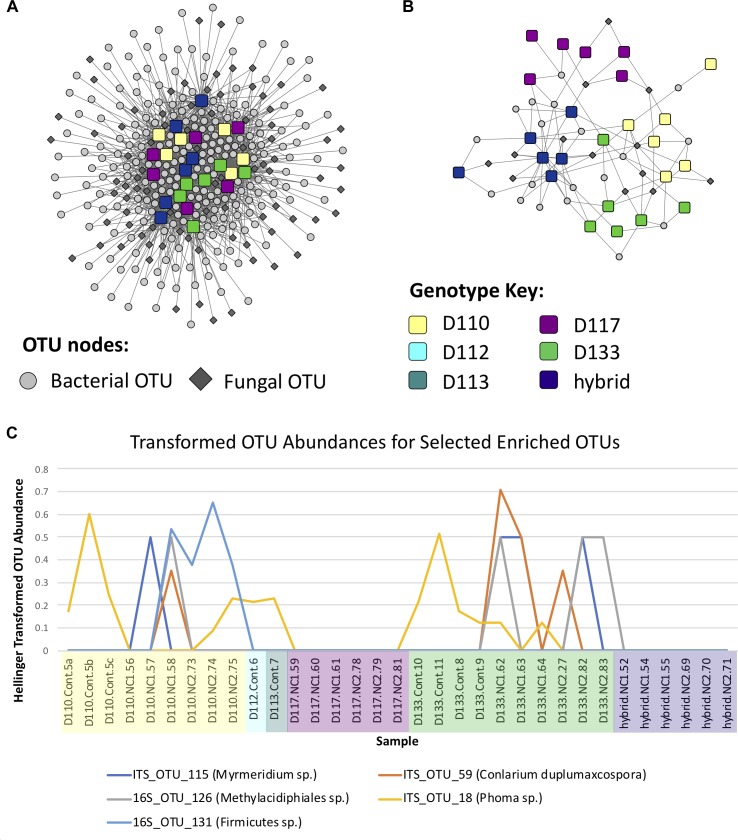
**(A)** Operational taxonomic unit (OTU)-sample enrichment networks representing the enrichment of OTUs in samples for Experiment 1, determined using the Fisher Exact test. **(B)** Subnetwork of the enrichment network involving genotype-specific OTUs, i.e., OTUs which were enriched in ≤ 2 genotypes and had a quotient ≥ 2 (see Materials and Methods and Supplementary Table S5). **(C)** Abundance line plots of selected OTUs which appear to be enriched in genotypes D110 and D133.

Bipartite fungal-bacterial networks show that soil origin and soil properties structured microbial communities more than host genotype, which is also reflected in sample correlations based on maximum spanning trees (Figure 4, Supplementary Figure S4, and Supplementary Table S4). Fisher’s Exact tests, investigating the enrichment of OTUs in genotypes separately within each soil type, reveal that the OTUs which are enriched in genotype samples vary based on the soil in which the genotype was grown (Supplementary Figure S5). This is contrary to expectations of a strong genotype effect, or evidence for vertical transmission of a pre-existing community. The enrichment bipartite network (Figure 5 and Supplementary Table S5) for Experiment 1 does not show any obvious grouping of genotypes based on their significant OTU associations. This indicates that OTUs that are enriched in a sample cannot be used to discriminate plant genotype. However, there are a few OTUs which do appear to be relatively genotype-specific. We calculated the quotient coefficient for each OTU by dividing the degree of each OTU node in the enrichment network by the total number of different genotypes that the OTU was enriched in Supplementary Table S5. Selecting only OTUs that were significantly associated with samples spanning ≤ 2 genotypes and with a quotient ≥ 2, one can see the effect of genotype grouping by considering only these “genotype-specific” OTUs (Figure 5B,C).

**FIGURE 6:**
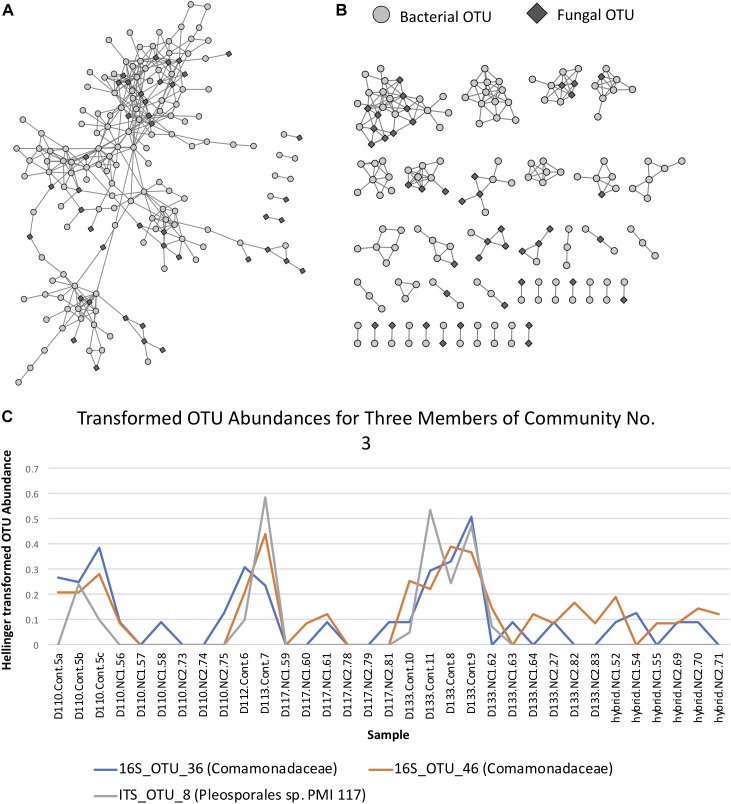
**(A)** Operational taxonomic unit (OTU) Pearson correlation network for Experiment 1, indicating the correlations between OTUs across samples. **(B)** Markov Cluster (MCL) community clusters of OTUs constructed from clustering of the network in Figure 6A. **(C)** Example line plot of 3 OTUs in MCL community 3, illustrating their correlated abundances.

Correlation analysis of the abundance of OTUs across samples resulted in a network with an obvious modular structure (Figure 6A). This was evident not only visually, but also quantitatively by looking at the distributions of various topology measures (Supplementary Table S6 and Supplementary Figure S6). The degree of a node indicates the number of neighbors it has in the correlation network. The degree distribution clearly indicates that most OTUs have a low degree, with a few OTUs representing hubs of high degree. The clustering coefficient of a node indicates the extent to which its neighbors are connected. The topological coefficient of a node indicates the extent to which a node shares neighbors with other nodes. The betweenness centrality of a node is an indicator of how many shortest paths pass through the node and is thus an indicator of “bottleneck nodes.” Betweenness is often high for nodes that form bridges between communities but are not within communities. The distributions of these network topology measures support the existence of a certain level of modular structure within the network, involving hub OTUs and communities. A small number of OTUs, even some that are not within communities, appear to serve as “bridges” between communities. Community construction based on Markov Cluster Algorithms (I = 2.5) resulted in 39 communities (Figure 6B and Supplementary Table S6). An example of the correlated abundances of OTUs within a community can be seen in Figure 6C. Communities varied in size within the smallest communities consisting of 2 OTUs and the largest community consisting of 25 OTUs. More than half of the communities (20 out of 39) consisted of both fungi and bacteria, with only one community lacking bacteria and 18 communities lacking fungi (Figure 6A and Supplementary Table S7). Taxa in the bacterial orders Rhizobiales, Chitinophagales, Cytophagales, and Burkholderiales were present in a majority of the networks. Ectomycorrhizal fungi, (i.e., *Hebeloma*, *Inocybe*, and *Thelephoraceae*) were included in three of the networks, each of which had more orders of bacterial taxa that differed than were shared (Supplementary Table S6). Many networks were comprised of endophytic fungi including species of *Atractiella, Fusarium*, Helotiales and bacteria, many of which have previously been reported and isolated from *Populus* roots (Shakya et al., 2013; Bonito et al. 2014, 2016).

### Experiment 2: Effect of Soil Origin on the Root Microbiome of a Single *Populus* Genotype

Differences in fungal root-associated communities were evident in samples of a single *Populus* genotype grown in soils collected from different locales (Figure 2). There were distinct differences in the fungal community composition detected between assayed soils, and even some of the most abundant taxa (i.e., *Atractiella* sp. nov, *Geopora* sp.1) were not in every soil (Figure 1). Only a single OTU (*Ilyonectria* sp.1) was detected in every sample. Likewise, there was much more variation in the root-associated bacterial communities of this single genotype grown in these eight different soils (Figure 2) than was observed for the different genotypes assayed in Experiment 1. Plants grown in the TN soils were more enriched in Micromonosporaceae and Flavisolibacter (Chitinophagaceae) compared to samples in Experiment 1 that were grown in soils from NC (Supplementary Figure S3). In particular, fungal and bacterial communities of samples grown in the soil TNPo1 were distinct from other samples. We calculated the average growth rate of *Populus* genotype D124 for plants grown in these different soil types (data not shown). A one-way ANOVA showed that these growth rates were not significantly different (*F* = 1.856, *p* = 0.129). Regarding fungal communities, PERMANOVA results support PCoA graphs and show that ∼68% of the sums of squares can be explained by the soil origin (*R*^2^ = 0.682, *p* = 0.0001, perm = 9999). Multivariate dispersions of sample groups did not differ statistically (*F* = 1.226, *p* = 0.344). Bacterial OTUs clustered according to soil origin (*R*^2^ = 0.519, *p* = 0.0001, perm = 9999), and no heterogeneity within group variances was detected (*F* = 0.362, *p* = 0.911).

### Indicator Species Analysis

Indicator species analysis identified twelve fungal taxa associated to specific soils, half of which were of ectomycorrhizal species including *Tuber mexiusanum, Hebeloma cavipes, Inocybe curvipes, Geopora* sp., *Pulvinula* sp, and Thelephoraceae sp. The root endophytic fungus *Atractiella rhizophila* was significantly associated with five of the sampled plots. Four bacterial taxa belonging to the Cytophagaceae, Chitinophagaceae, Acidimicrobiales, and Hyphomonadaceae were identified by species indicator analysis (Table 1) to be statistically associated (*P* < 0.05) to a particular soil.

### Impact of Soils on Fungal and Bacterial Networks

**FIGURE 7:**
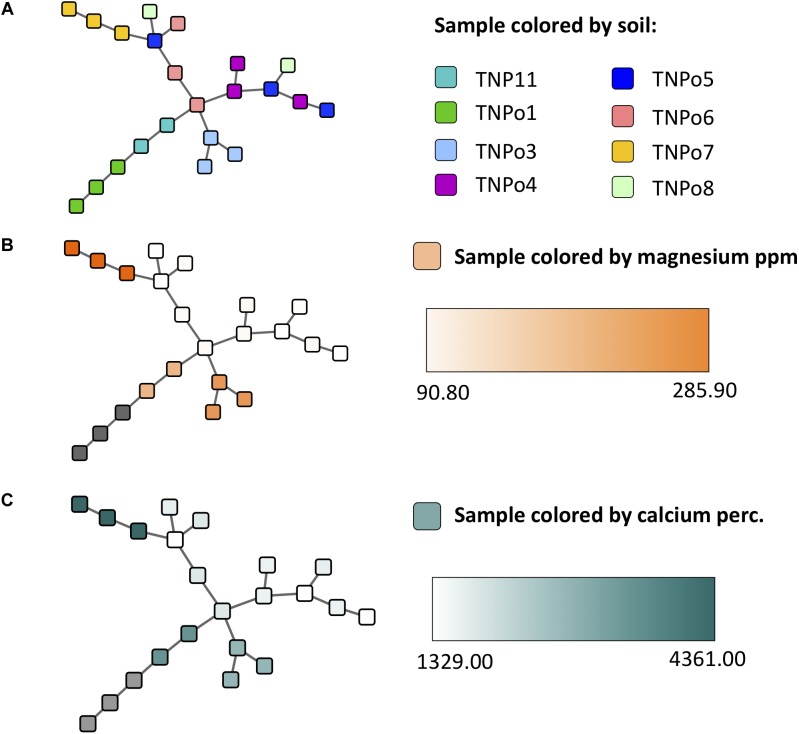
Experiment 2 (soil origin) sample similarity MSTs (constructed using the Proportional Similarity metric) representing similarity of samples based on their operational taxonomic unit (OTU) content, colored by **(A)** soil, **(B)** magnesium ppm of soil, and **(C)** calcium content of soil.

**FIGURE 8:**
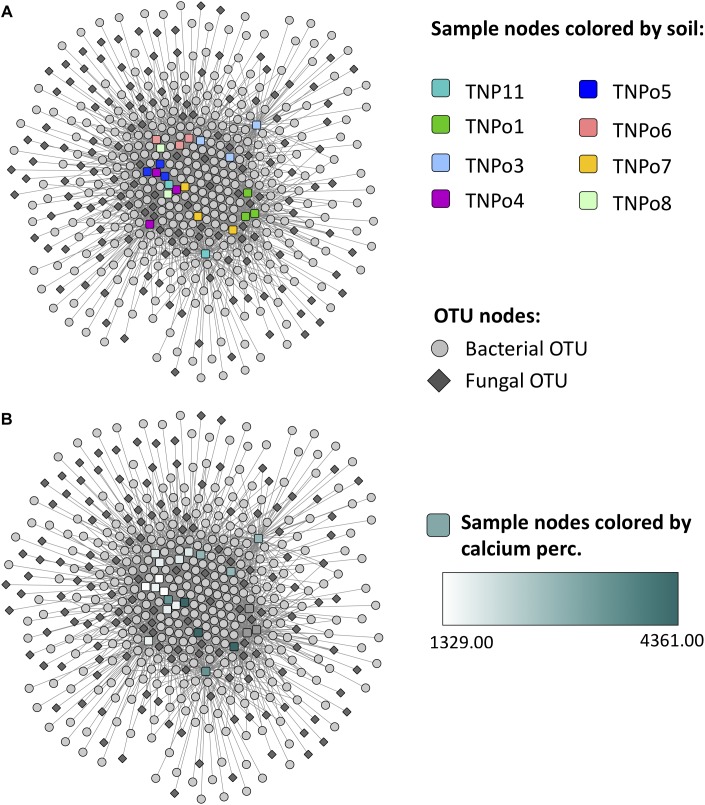
Operational taxonomic unit (OTU)-sample enrichment networks representing the enrichment of OTUs in samples for Experiment 2: Soil origin. An OTU node is connected to a sample node via an edge if the OTU is significantly enriched in that sample. Sample nodes are colored by **(A)** soil and **(B)** calcium content.

**FIGURE 9:**
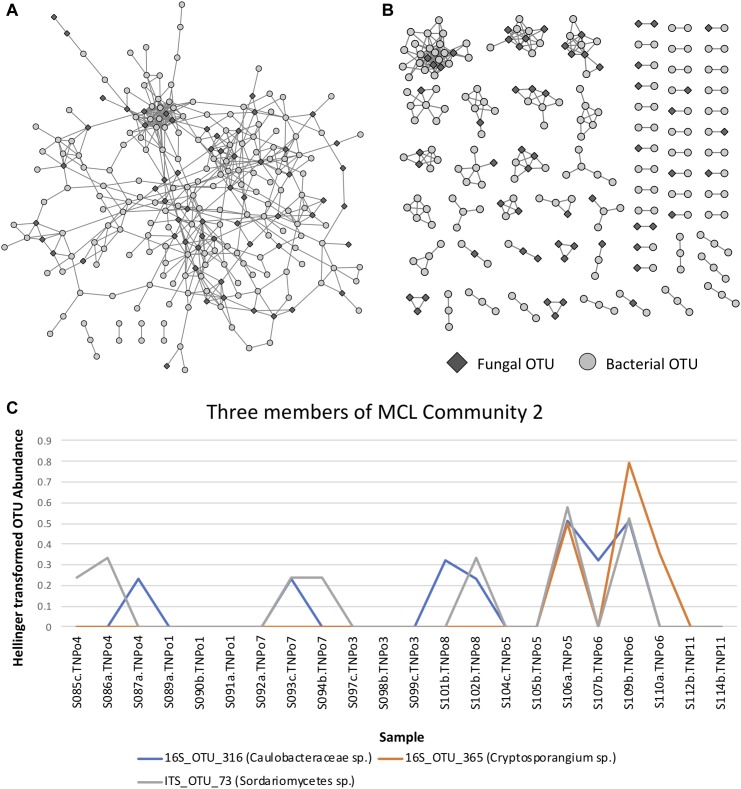
**(A)** Operational taxonomic unit (OTU) Pearson correlation network for Experiment 2 (soil origin), indicating the correlations between OTUs across samples. **(B)** Markov Cluster (MCL) community clusters of OTUs constructed from clustering of the network in Figure 9A. **(C)** Example line plot of 3 OTUs in MCL community 2, illustrating their correlated abundances.

Maximum spanning tree networks representing sample similarity based on OTU content revealed that root-associated microbial communities are largely structured by soil (Figure 7). Soil characteristics such as calcium and magnesium appeared to have a large influence on the structure of fungal-bacterial networks, as one can see that samples tend to segregate based on these variables (Figure 7B,C). OTU enrichment bipartite networks also tend to group samples by soil (Figure 8A) and segregation of sample nodes appears to follow a calcium gradient (Figure 8B). Correlation analysis of OTU abundances across samples resulted in a network with a modular structure (Figure 9A). Again, topological analysis of the network through various network topology measures quantitatively supports the existence of some modularity, and thus, potential communities (Supplementary Figure S7 and Supplementary Table S8). Community construction using MCL (*I* = 2.5) resulted in 66 communities (Figure 9B). Communities represent groups of OTUs with correlated abundances across samples (For example, Figure 9C). OTU communities varied in size, with the largest community consisting of 24 members and the smallest communities consisting of 2 members. Of the 66 communities, 28 consisted of both bacteria and fungi, 33 consisted of only bacteria and 5 consisted of only fungi (Supplementary Table S9). *Atractiella* was associated with one fungal-bacterial network composed of *Atractiella* and the bacterium Chitinophagaceae. Arbuscular mycorrhizal fungi were included in three of the networks. While some ectomycorrhizal taxa such as *Tuber* and *Tomentella* were shared between networks, most ectomycorrhizal taxa overrepresented in fungal-bacterial networks belonged to different sub-networks.

**FIGURE 10:**
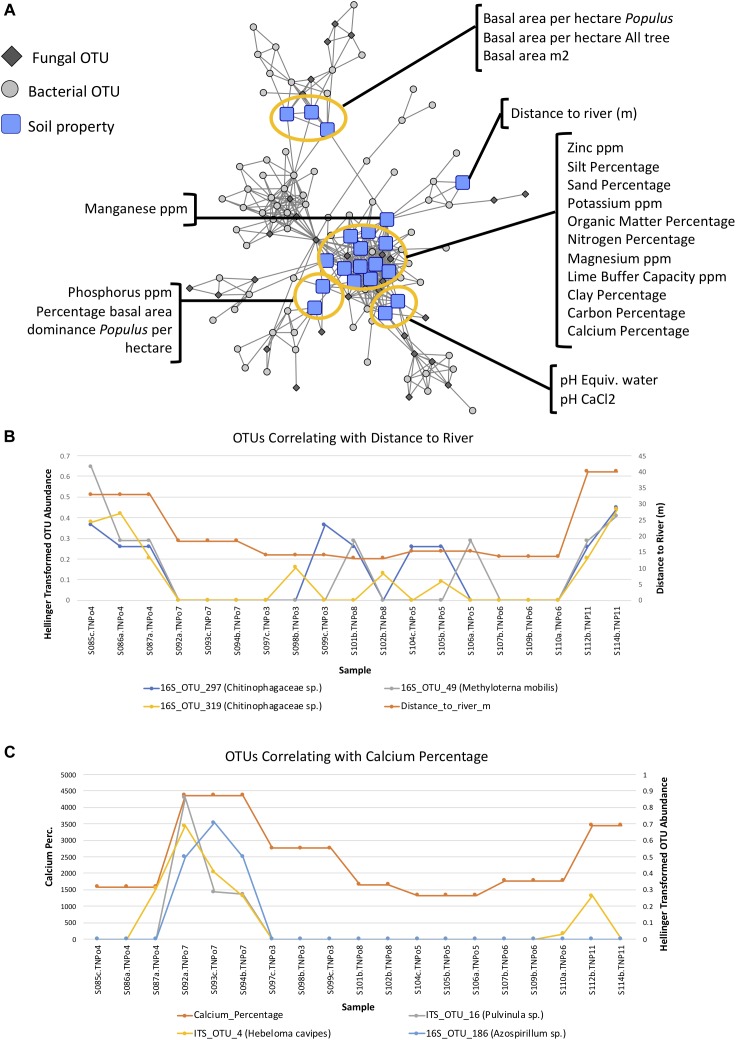
**(A)** Pearson correlation network of operational taxonomic unit (OTU) communities and soil physicochemical/environmental parameters at a threshold of | Pearson|≥ 0.6, indicating the correlation between the various soil properties with OTU abundance across samples in Experiment 2: Soil origin. **(B)** Example of 3 OTUs whose abundances correlate with distance from the river. **(C)** Example of three OTUs whose abundances correlate with calcium content of soil.

Certain soil properties correlated with one another (Supplementary Figure S8) and with certain OTU abundances and appear to be potentially driving communities (Figure 10A). For example, distance to the river is correlated with two Chitinophagaceae 16S OTUs in MCL community 32 as well as an *Methylotenera mobilis* OTU in community 20 (Figure 10B), and calcium percentage is correlated with OTUs of ECM fungi (e.g., *Hebeloma cavipes*, *Pulvinua* sp.) in MCL community 3 (Figure 10C), indicating potential drivers of these communities.

## Discussion

Plant microbiomes are diverse and complex in structure and function. In this study, we assessed the impact of plant genotype and soil on root microbiome of *Populus* using natural soils as inoculum and plant bioassay experiments in a common garden design. Results show that bacterial diversity in the *Populus* rhizobiome is many times higher than fungal diversity. Genetic variation of plant hosts is known to be important to herbivore and pathogen resistance (Keith and Mitchell-Olds, 2013), and host genetics have been shown to be important in plant selection for mutualistic ectomycorrhizal fungi and their functioning (Gehring et al., 2017). We found that the impact of host genotype was dependent on soil, and that the effect was at the community level, with no apparent impact on specific ITS or 16S OTUs. For example, the top 20 most abundant fungi were found on all genotypes, and no taxon responding to host genotype was observed across all sample replicates of any genotype. Additionally, indicator species analysis failed to detect any OTUs of ITS or 16S statistically associated to a specific *Populus* genotype. Similar results were obtained for prokaryotes, with *Niastella*, Micromonosporineae, and *Bradyrhizobium* being particularly well represented in the *Populus* rhizobiome across treatments. In contrast, despite the fact that many fungal root endophytes of *Populus* appear to have widespread distributions, soil origin and soil physicochemical characteristics explained most of the measurable variation in fungal and bacterial communities and multi-kingdom bipartite networks. In particular, fungal-bacterial networks clustered in relation to geographic location, soil texture and soil phosphorus level. These results indicate that microbial taxa are not evenly distributed across the landscape, and plant microbiome composition is largely influenced by edaphic factors and local environment.

*Populus* can establish both ectomycorrhizal and arbuscular mycorrhizal symbiosis (Vozzo and Hacskaylo, 1974; Lodge, 1989). We detected several ectomycorrhizal fungi in *P. deltoides* and *P. deltoides* x *P. trichocarpa* hybrid genotypes include *Inocybe* spp, *Hebeloma* spp., Pezizales spp., *Geopora* spp. and Thelephoraceae. Arbuscular mycorrhizal fungi were detected in the diverse soil treatments, including Glomeraceae and Paraglomeraceae, as previously shown (Bonito et al., 2014). No impact of genotype or soil was observed for arbuscular mycorrhizal fungi. This result was expected since, ecologically, most arbuscular mycorrhizal fungi are considered to be host-generalists. *Populus deltoides* associates with a variety of fungal symbionts, rather than specializing with specific taxa. Despite the presence of arbuscular and ectomycorrhizal fungi, our data demonstrate that the rhizobiome of *Populus* is dominated by facultative endophytes (∼85% OTUs), most of which appear to be culturable and have saprotrophic activity. For example, *Atractiella rhizophila* was first described from *Populus* and detected as an abundant OTU in natural habitats through molecular assessments (Gottel et al., 2011; Bonito et al., 2017). We found this taxon to be dominant (in terms of relative sequence abundance) in five of the eight soils used in the bioassay experiments, and this taxon was present in soils from both North Carolina and Tennessee. Other endophytic fungi commonly detected in *Populus* include *Ilyonectria* spp.(= *Cylindrocarpon*), *Fusarium oxysporum*, *Phoma herbarum*, Chaetothyriales sp., and *Sebacina* sp. Ecologically, many of these genera are regarded as plant pathogens (e.g., *Fusarium* spp., *Ilyonectria* spp., *Phoma* spp.), yet there is no indication that these specific fungi are pathogenic to *Populus*. In fact, some species within these genera are even reported as plant growth-promoting fungi (Hamayun et al., 2009). For these reasons, we were cautious about trying to overclassify the function(s) of these OTUs. The ability of *Populus* to host fungi in high abundance that are considered to be generalist pathogens to other plants may offer a competitive advantage to *Populus* in certain environments. Further, virulence of potential pathogens could be dictated by multi-partner interactions and environmental conditions that may be detectable through network analyses (Hersh et al., 2012). Further, disease susceptibility can vary between hosts and may interact with host genotype. A large number of OTUs and groups of endophytes on *Populus* are similar to those endophytic communities found in the rhizosphere of non-mycorrhizal *Microthlaspi* species (R Core Team, 2018). We hypothesize that these fungi are widespread in the rhizosphere of other plant species. Although some of the fungal taxa have been studied in pairwise situations with host plants (Bonito et al., 2017), the functioning of most of these species in multi-species interactions and plant rhizospheres is still relatively unexplored (Vélez et al., 2017).

Network analyses provide a powerful tool for distilling information from large datasets to generate hypotheses on keystone taxa that impact the assembly and functioning of multi-kingdom microbiome communities (Hoppe et al., 2014). Multi-Kingdom hypotheses generated through network analyses can then be tested directly through various experimental approaches, including through baiting or manipulative experiments (Ghodsalavi et al., 2017). Such an approach was used to study the phyllosphere microbiome of *Arabidopsis thaliana*. Manipulation of the pathogenic oomycete *Albugo*, a “hub” taxa with high inter-connectivity with other taxa, was shown to have dramatic impacts on the assembly of epiphytic and endophytic bacterial communities (Agler et al., 2016). Constructed fungal-bacterial communities in our study helped to identify hub taxa, having a high degree of connectivity (Supplementary Figure S5), which included a higher number of bacteria (e.g., *Leptospira, Cytophagaceae, Chitinophagaceae, Micromonosporaceae*) compared to fungi (e.g., *Geopora*, *Fusarium*, Sordariomycete) (Supplementary Table S8), and a few bridge taxa that have a high betweenness distribution. Network analysis also helped to identify microbial taxa that correlate with particular plant genotypes, soils, and soil characters (e.g., % Ca) (Figure 5–10). Hub and bridge taxa did not overlap between the two conducted experiments, possibly due to the fact that there was no overlap in plant genotype or soils between the two experiments. Over-represented taxa in microbial networks were disproportionately organisms representing different ecological guilds (e.g., saprotrophs, pathogens, ectomycorrhizal, arbuscular mycorrhizal); however, the taxonomic identification of many of these taxa is stuffiest to make strong ecological predictions. Although many indicator fungal species were ectomycorrhizal taxa, most ectomycorrhizal taxa belonged to different networks, comprised of different 16S OTUs, indicating that the rhizobiome niche may be partitioned in between different niches comprised of alternate sets of microbial consortia. Identifying and understanding the function of keystone microbial species in plant microbiomes offers new approaches for managing plant microbiomes for health and sustainability.

## Conclusion

Data from two experiments provide assessments on impacts of soil and *Populus’* genotype on fungal and bacterial communities comprising the rhizobiome. The impact of host genotype on the composition of root microbial communities was stronger for bacteria than for fungi, and was dependent upon the particular soil the plants were grown in. In contrast, soil origin was shown to be a significant factor driving the composition of fungal and bacterial communities and networks in *Populus* roots, which was previously reported (Bonito et al., 2014; Cregger et al., 2018). Soil physicochemical parameters including texture and calcium concentration contribute to this soil effect, but other unmeasured factors likely play a role in the *Populus* root microbiome assembly. Importantly, uninoculated control plants were distinct from communities on treatment plants indicating that results are not due to methodological artifacts. Finally, network analyses identified fungal-bacterial consortia that were statistically overrepresented in the dataset, providing hypotheses for testing and a framework for expanding this research from mesocosms to the field.

## Data Availability

The 454 sequences have been submitted to GenBank’s sequence read archive under the study accession number SRP106691.

## Author Contributions

GB, RV, KH, CS, and K-HC contributed to the experimental design and carrying out the experiments. GB, GMNB, PJ, DW, DJ carried out the data and statistical analysis. All authors contributed to the writing of this manuscript.

## Conflict of Interest Statement

The authors declare that the research was conducted in the absence of any commercial or financial relationships that could be construed as a potential conflict of interest.

## Footnotes

1 http://aesl.ces.uga.edu/

2 http://www.cpan.org/

3 http://www.cpan.org/

## Supplementary Material

Figure S1 Rarefaction curves for ITS and 16S rDNA from Experiments 1 and 2.

Figure S2 Contingency table used in the calculation of Fisher Exact Tests. Each mij is entry ij of an operational taxonomic unit (out) matrix/binned OTU matrix M.

Figure S3 Boxplots showing the relative abundance of fungi and bacteria across different hierarchical classification for the two experiments. **(A)** Fungi across different soils (Experiment 2: Soil origin); **(B)** Fungi across different Populus genotypes; **(C)** Bacteria across different soils (Experiment 2: Soil origin); **(D)** Bacteria across different Populus genotypes.

Figure S4 Raw bipartite networks representing the occurrence of operational taxonomic units (OTUs) within samples for Experiment 1: Plant genotype. Sample nodes are colored by **(A)** genotype and **(B)** soil. Samples clearly separate by soil and not be genotype.

Figure S5 Enrichment of operational taxonomic units (OTUs) in genotypes, within each soil. OTU nodes are connected to genotype nodes via colored “soil specific” edges. An edge between an OTU and a genotype represents that the OTU is enriched in that genotype within the soil denoted by the edge color. One can clearly see that the enrichment of OTUs in genotypes is very heavilty influenced by the soil in which the genotype was grown.

Figure S6 Distributions of selected network topology measures for the Experiment 1 operational taxonomic unit (OTU) correlation network.

Figure S7 Distributions of selected network topology measures for the Experiment 2 operational taxonomic unit (OTU) correlation network.

Figure S8 Correlation network maximum spanning tree of soil physicochemical and environmental parameters.

Table S1 Characteristics of soils used in this study.

Table S2 Primer sequence information for 454 primers used in this study.

Table S3 Unique fungal operational taxonomic units (OTUs) recovered from uninoculated control plants.

Table S4 Degree of each operational taxonomic unit (OTU) in the Experiment 1 bipartite network, as well as the number of different genotypes the OTU is found in.

Table S5 Degree of each operational taxonomic unit (OTU) in the Experiment 1 enrichment network, the number of different genotypes the OTU is significantly enriched in, as well as the quotient of degree/#genotypes. The quotient gives an indication of how genotype-specific the OTU is.

Table S6 Operational taxonomic units (OTUs) assigned to Markov Cluster (MCL) communities for Experiment 1, as well as various network topology measures each OTU node in the pearson correlation network the communities were constructed from.

Table S7 Summary of the sizes of Markov Cluster (MCL) communities for Experiment 1, as we as the number of fungi vs bacterial members.

Table S8 Operational taxonomic units (OTUs) assigned to Markov Cluster (MCL) communities for Experiment 2, as well as various network topology measures each OTU node in the pearson correlation network the communities were constructed from.

Table S9 Summary of the sizes of Markov Cluster (MCL) communities for Experiment 2, as we as the number of fungi vs bacterial members.

FILE S1 Assessments of arbuscular mycorrhizal fungal diversity.

FILE S2 Cytoscape session containing the networks for Experiment 1.

FILE S3 Cytoscape session containing the networks for Experiment 2.
