# Supplementary figures and images for: Fungal-Bacterial Networks in the Populus Rhizobiome Are Impacted by Soil Properties and Host Genotype
Source: Front Microbiol. 2019 Mar 29;10:481. doi: 10.3389/fmicb.2019.00481 (PMC6450171; doi:10.3389/fmicb.2019.00481)

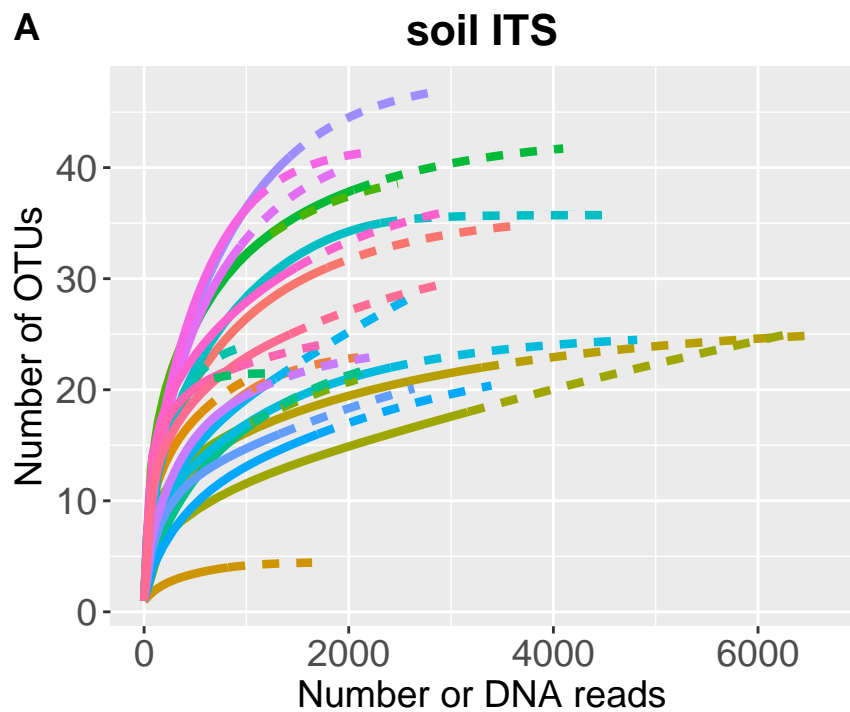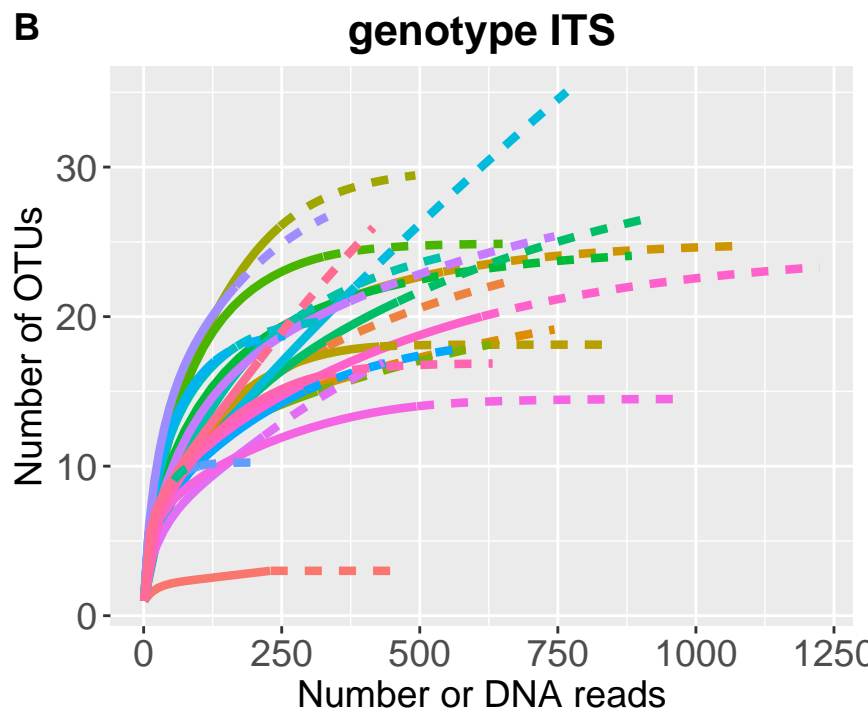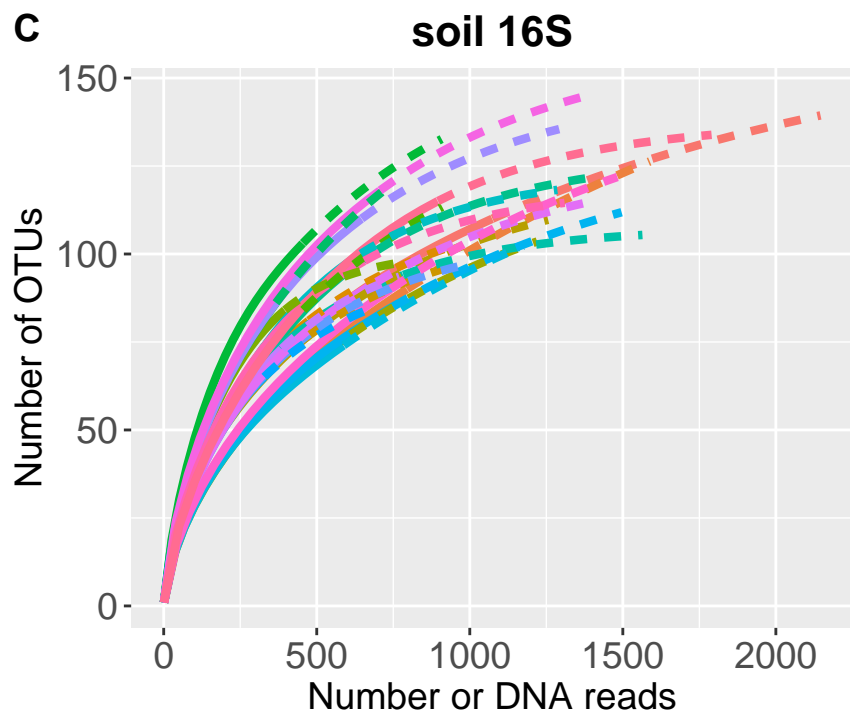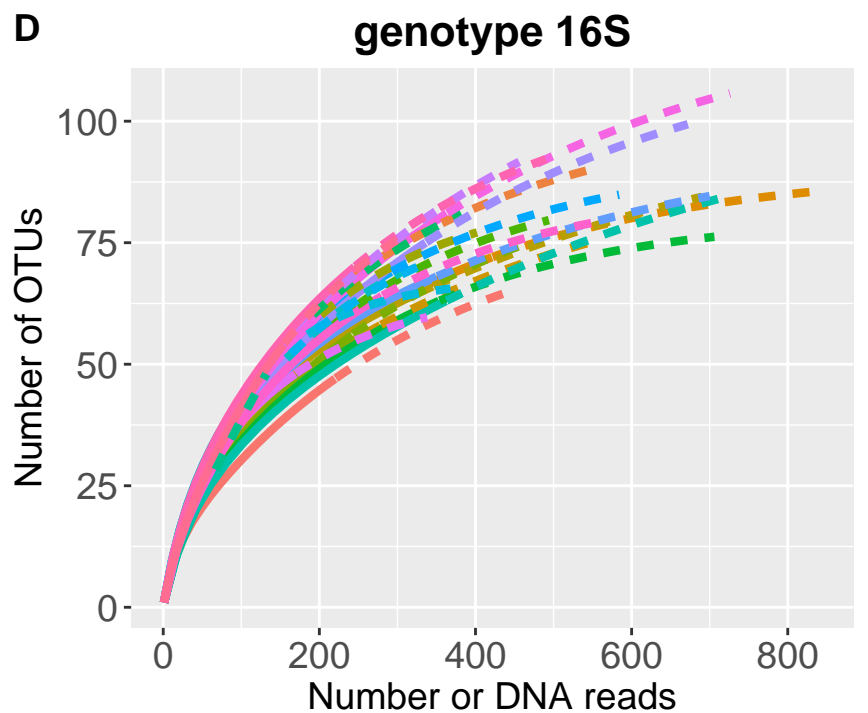

— interpolated    - - - extrapolated

Supplement: Figure S1 — Rarefaction curves for ITS and 16S rDNA from Experiments 1 and 2. [file Image_1.pdf]

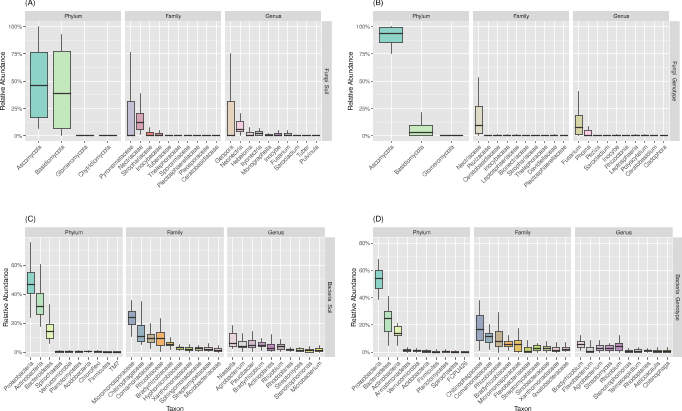

Supplement: Figure S3 — Boxplots showing the relative abundance of fungi and bacteria across different hierarchical classification for the two experiments. (A) Fungi across different soils (Experiment 2: Soil origin); (B) Fungi across different Populus genotypes; (C) Bacteria across different soils (Experiment 2: Soil origin); (D) Bacteria across different Populus genotypes. [file Image_3.tif]

**A**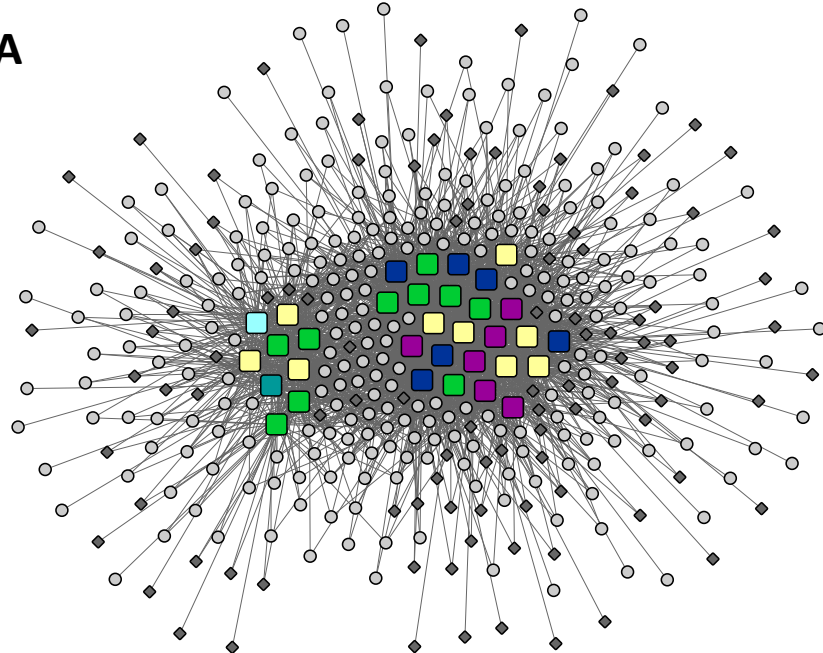**Genotype Key:**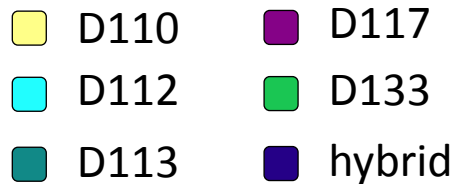**OTU nodes:**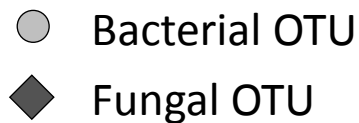**B**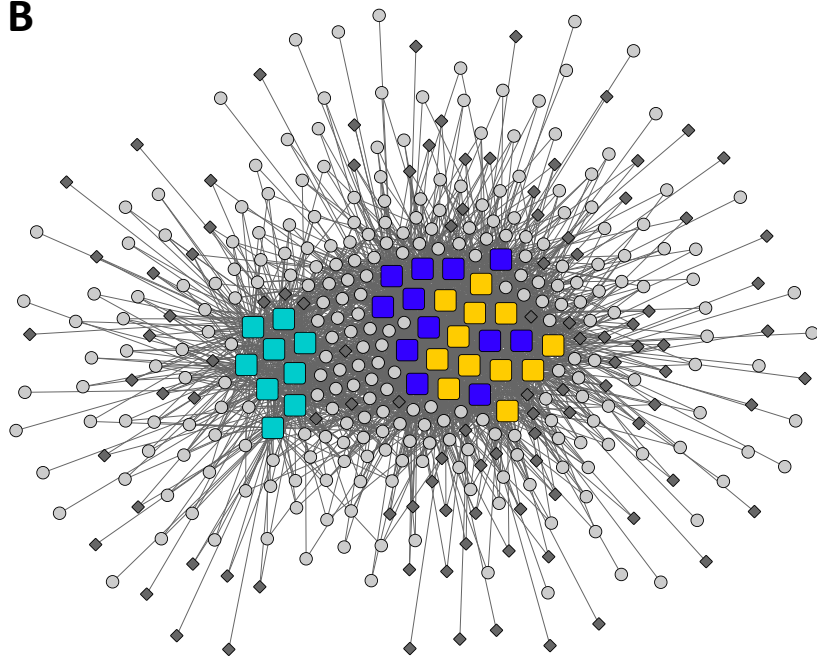**Soil Key:**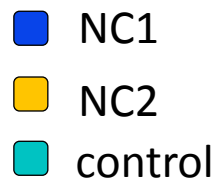

Supplement: Figure S4 — Raw bipartite networks representing the occurrence of operational taxonomic units (OTUs) within samples for Experiment 1: Plant genotype. Sample nodes are colored by (A) genotype and (B) soil. Samples clearly separate by soil and not be genotype. [file Image_4.pdf]

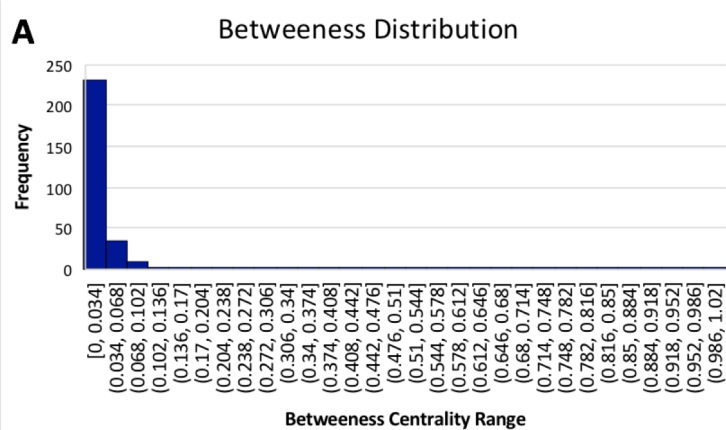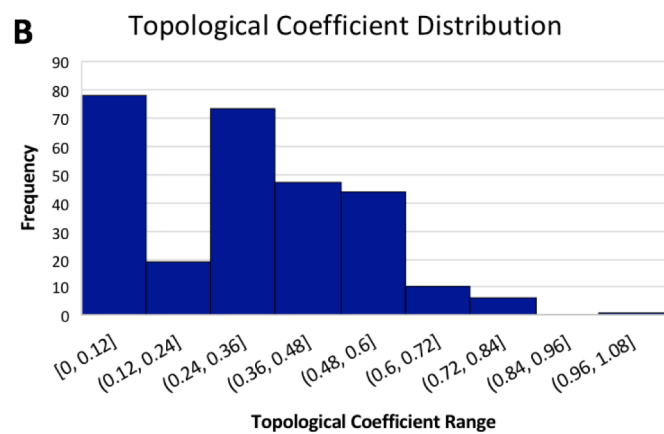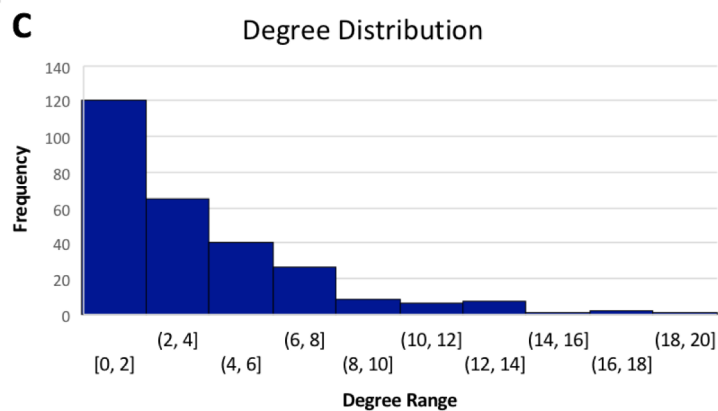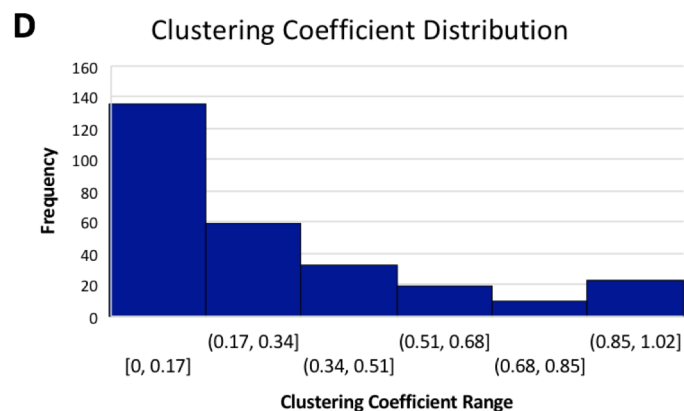

Supplement: Figure S7 — Distributions of selected network topology measures for the Experiment 2 operational taxonomic unit (OTU) correlation network. [file Image_7.pdf]

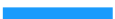 Negative correlation

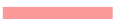 Positive correlation

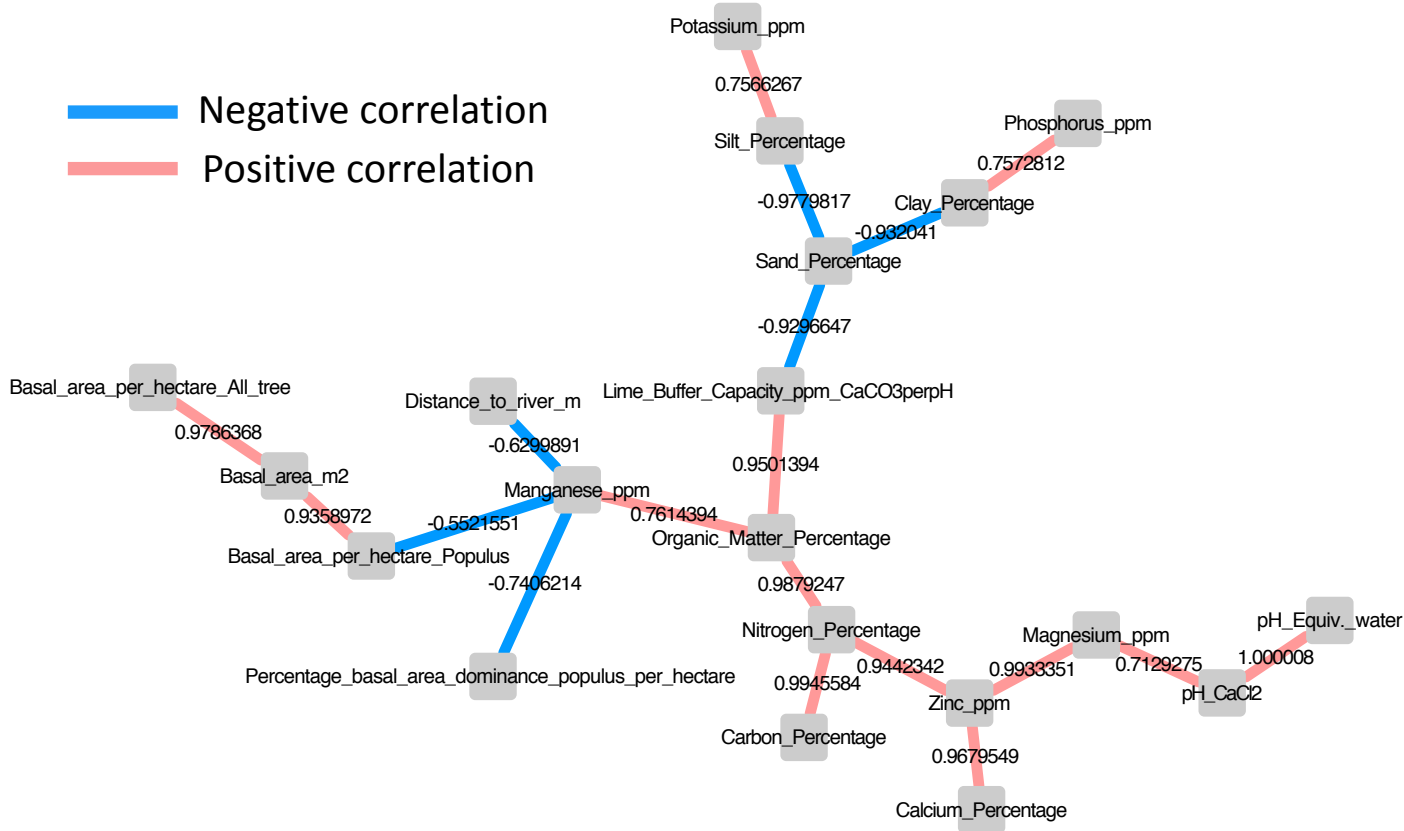

Supplement: Figure S8 — Correlation network maximum spanning tree of soil physicochemical and environmental parameters. [file Image_8.pdf]
