# Supplementary material for: Fungal-Bacterial Networks in the Populus Rhizobiome Are Impacted by Soil Properties and Host Genotype
Source: Front Microbiol. 2019 Mar 29;10:481. doi: 10.3389/fmicb.2019.00481 (PMC6450171; doi:10.3389/fmicb.2019.00481)
Supplement: Figure S2 — Contingency table used in the calculation of Fisher Exact Tests. Each mij is entry ij of an operational taxonomic unit (out) matrix/binned OTU matrix M. [file Image_2.pdf]

	Sample y	$\neg$Sample y
OTU x	m_{xy}	$\sum_{j \neq y} m_{xj}$
$\neg$OTU x	$\sum_{i \neq x} m_{iy}$	$\sum_{i \neq x} \sum_{j \neq y} m_{ij}$
