# Supplementary material for: Fungal-Bacterial Networks in the Populus Rhizobiome Are Impacted by Soil Properties and Host Genotype
Source: Front Microbiol. 2019 Mar 29;10:481. doi: 10.3389/fmicb.2019.00481 (PMC6450171; doi:10.3389/fmicb.2019.00481)
Supplement: Figure S5 — Enrichment of operational taxonomic units (OTUs) in genotypes, within each soil. OTU nodes are connected to genotype nodes via colored “soil specific” edges. An edge between an OTU and a genotype represents that the OTU is enriched in that genotype within the soil denoted by the edge color. One can clearly see that the enrichment of OTUs in genotypes is very heavilty influenced by the soil in which the genotype was grown. [file Image_5.pdf]

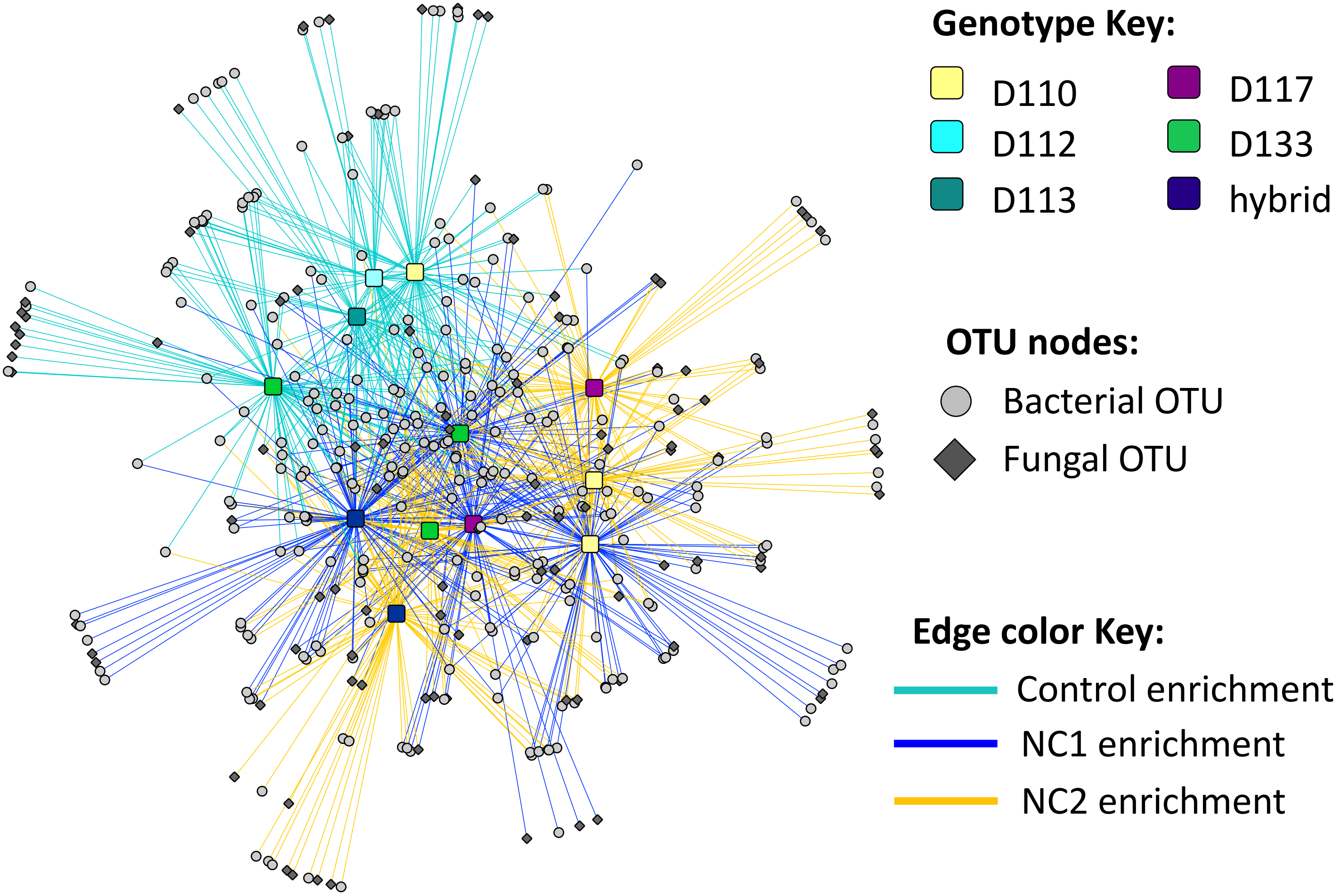

Genotype Key:
D110
D117
D112
D133
D113
hybrid
OTU nodes:
Bacterial OTU
Fungal OTU
Edge color Key:
Control enrichment
NC1 enrichment
NC2 enrichment
