# Supplementary material for: Fungal-Bacterial Networks in the Populus Rhizobiome Are Impacted by Soil Properties and Host Genotype
Source: Front Microbiol. 2019 Mar 29;10:481. doi: 10.3389/fmicb.2019.00481 (PMC6450171; doi:10.3389/fmicb.2019.00481)
Supplement: Figure S6 — Distributions of selected network topology measures for the Experiment 1 operational taxonomic unit (OTU) correlation network. [file Image_6.pdf]

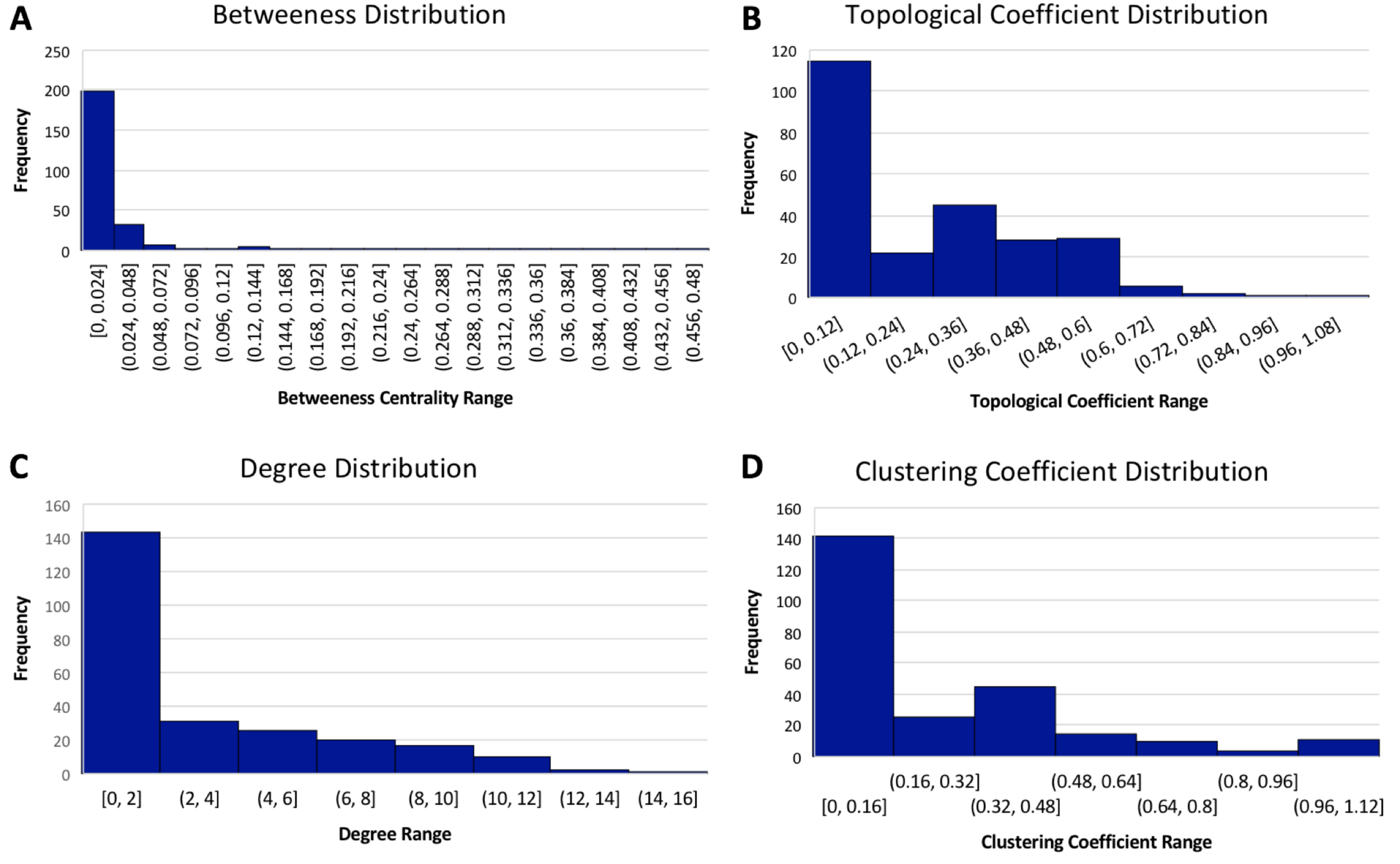
A
Betweeness Distribution
Frequency
Betweeness Centrality Range
[0, 0.024]
(0.024, 0.048]
(0.048, 0.072]
(0.072, 0.096]
(0.096, 0.12]
(0.12, 0.144]
(0.144, 0.168]
(0.168, 0.192]
(0.192, 0.216]
(0.216, 0.24]
(0.24, 0.264]
(0.264, 0.288]
(0.288, 0.312]
(0.312, 0.336]
(0.336, 0.36]
(0.36, 0.384]
(0.384, 0.408]
(0.408, 0.432]
(0.432, 0.456]
(0.456, 0.48]
B
Topological Coefficient Distribution
Frequency
Topological Coefficient Range
[0, 0.12]
(0.12, 0.24]
(0.24, 0.36]
(0.36, 0.48]
(0.48, 0.6]
(0.6, 0.72]
(0.72, 0.84]
(0.84, 0.96]
(0.96, 1.08]
C
Degree Distribution
Frequency
Degree Range
[0, 2]
(2, 4]
(4, 6]
(6, 8]
(8, 10]
(10, 12]
(12, 14]
(14, 16]
D
Clustering Coefficient Distribution
Frequency
Clustering Coefficient Range
[0, 0.16]
(0.16, 0.32]
(0.32, 0.48]
(0.48, 0.64]
(0.64, 0.8]
(0.8, 0.96]
(0.96, 1.12]
