# Supplementary material for: Fungal-Bacterial Networks in the Populus Rhizobiome Are Impacted by Soil Properties and Host Genotype
Source: Front Microbiol. 2019 Mar 29;10:481. doi: 10.3389/fmicb.2019.00481 (PMC6450171; doi:10.3389/fmicb.2019.00481)
Supplement: FILE S1 — Assessments of arbuscular mycorrhizal fungal diversity. [file Data_Sheet_1.docx]

**Supplementary Files S1 - Assessments of arbuscular mycorrhizal fungal diversity**

Arbuscular mycorrhizal fungi (AMF) are important components for the *Populus* rhizobiome [(Lodge, 1989)](https://paperpile.com/c/JpurLD/1HTH), thus, additional studies were conducted to better assess and voucher their diversity on *Populus* through molecular cloning from roots, and by soil spore extractions and Sanger sequencing for molecular taxonomic determination. Because AMF are obligate biotrophs, isolates must be kept alive with a compatible plant host. Sorghum is one host that been extensive used to maintain genetic lines of AMF [(Selvakumar *et al.*, 2016)](https://paperpile.com/c/JpurLD/DezI). In order to maintain and increase AMF propagules pot cultures of sorghum were inoculated with AMF spores extracted from *Populus* bioassay plant roots that had been grown in NC1 and NC2 soils (two plants from each *Populus* genotypes used in Experiment 1 and a previous published study - Bonito *et al.*, 2014).

Harvested *Populus* roots were shook to remove loose soil. AMF were extracted from roots by immediately grinding with 500 ml of water in a Waring blender. The water-root-soil mix was transferred and sieved through two meshes (500 µm and 53 µm sieves). The material caught on the 53 m sieve was transferred to a 50 ml centrifuge tube and water was added to bring up the volume to 15 ml. We added 10 ml 60% sucrose to the tube and centrifuged for 3 mins at 2000 rpm. After the centrifugation, the upper layer of liquid was transferred on to the 38 µm sieve and washed with tap water for 1 min. The material was transferred to a Petri dish. Spores were sorted morphologically and were used to inoculate 18 sorghum pots. Sorghum plants were grown to maturity, after which AMF spores were extracted from their roots as described above for *Populus*.

Up to four spores were picked into a 0.2 ml PCR tube and crushed by needles under dissecting microscope. The DNA extraction was described by [(Lee *et al.*, 2006)](https://paperpile.com/c/JpurLD/k5uI). A nested PCR approach was used to amplify the rSSU region of AMF. For the 1st PCR reaction, each 25 μl reaction mixture included 4 μl (1 mM) dNTPs with 2.5 μl BSA (10 mg/ml), 1.25 μl of NS1 and NS4 primers [(White *et al.*, 1990)](https://paperpile.com/c/JpurLD/9G2R) (10 μM), 2 μl of DNA template, and 11.35 μl of water. The thermocycler condition of the 1st PCR reaction was: 95°C for 10 mins; 20 cycles of 95°C for 1 min, 52°C 30s with a touchdown setting that decrease 0.5 °C every cycle, 72°C for 1 min; and 72°C for 7 mins. Each 25 μl reaction mixture of the 2nd PCR reaction included 4 μl (1 mM) dNTPs with 2.5 BSA μl (10 mg/ml), 1.25 μl of AML1 and AML2 primers [(Lee *et al.*, 2008)](https://paperpile.com/c/JpurLD/ySLN) (10 μM), 1 μl of PCR product from the 1st PCR reaction, and 12.35 μl of water. The thermocycler condition of the 2nd PCR reaction was: 95°C for 3 mins; 30 cycles of 95°C for 1 min, 50°C for 30s, 72°C for 1 min; and 72°C for 10 mins). Exonuclease and Antarctive phosphatase enzymes were used to clean PCR products prior to Sanger sequencing on an ABI3700 capillary sequencer (Applied Biosystems). Obtained sequences were quality checked, trimmed, and assembled at 99% similarity in Sequencher v4.5 (Gene Codes Corporation, Ann Arbor, MI). Sequences were then aligned to reference sequences in MUSCLE [(Edgar, 2004)](https://paperpile.com/c/JpurLD/AmHj) and their phylogenetic placement was estimated in RaxML [(Stamatakis *et al.*, 2008)](https://paperpile.com/c/JpurLD/kXie).

**References**

[**Edgar RC**. **2004**. MUSCLE: multiple sequence alignment with high accuracy and high throughput. *Nucleic acids research* **32**: 1792–1797.](http://paperpile.com/b/JpurLD/AmHj)

[**Lee J, Lee S, Young JPW**. **2008**. Improved PCR primers for the detection and identification of arbuscular mycorrhizal fungi. *FEMS microbiology ecology* **65**: 339–349.](http://paperpile.com/b/JpurLD/ySLN)

[**Lee J, Park S-H, Eom A-H**. **2006**. Molecular identification of arbuscular mycorrhizal fungal spores collected in Korea. *Mycobiology* **34**: 7–13.](http://paperpile.com/b/JpurLD/k5uI)

[**Lodge DJ**. **1989**. The influence of soil moisture and flooding on formation of VA-endo- and ectomycorrhizae in Populus and Salix. *Plant and soil* **117**: 243–253.](http://paperpile.com/b/JpurLD/1HTH)

[**Selvakumar G, Krishnamoorthy R, Kim K, Sa T**. **2016**. Propagation technique of arbuscular mycorrhizal fungi isolated from coastal reclamation land. *European journal of soil biology* **74**: 39–44.](http://paperpile.com/b/JpurLD/DezI)

[**Stamatakis A, Hoover P, Rougemont J**. **2008**. A Rapid Bootstrap Algorithm for the RAxML Web Servers. *Systematic biology* **57**: 758–771.](http://paperpile.com/b/JpurLD/kXie)

[**White TJ, Bruns T, Lee S, Taylor JL, Others**. **1990**. Amplification and direct sequencing of fungal ribosomal RNA genes for phylogenetics. *PCR protocols: a guide to methods and applications* **18**: 315–322.](http://paperpile.com/b/JpurLD/9G2R)


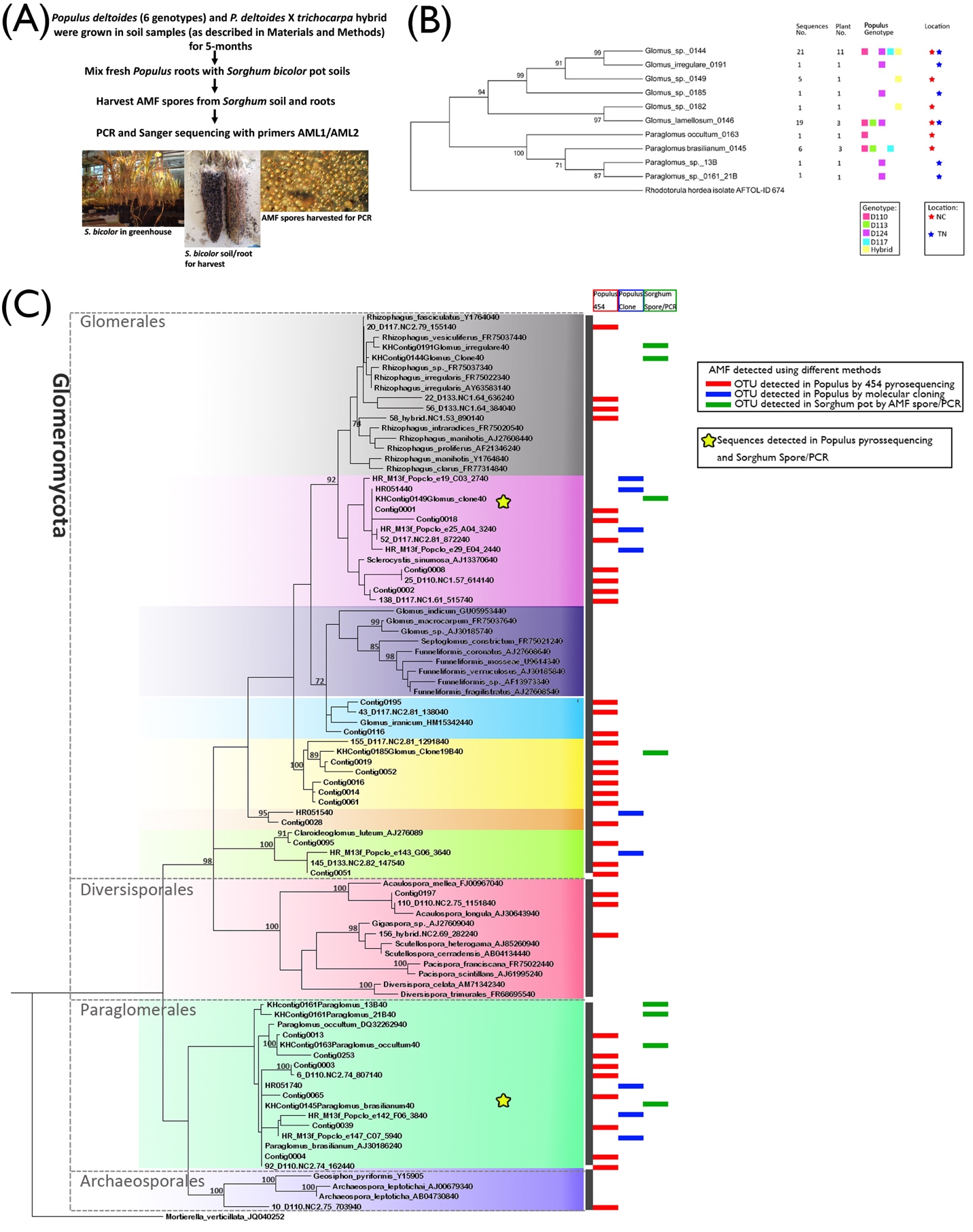


**Supplementary Files S1**

Arbuscular Mycorrhizal Fungi (AMF) are important components of *Populus* rhizobiome (Bonito et al., 2014), so attempts were made to retain AM fungal vouchers using *Sorghum* pot cultures. (A) Workflow of *Populus* root*-Sorghum* pot culture and AMF detection. (B) The phylogeny based on 10 AMF SSU OTUs detected in *S. bicolor* pot cultures. (C) The Phylogeny of AMF SSUs detected in *Populus* bioassay roots by 454 pyrosequencing, in *Populus* bioassay roots by molecular cloning, and in *Sorghum* pot (in either soil or roots) cultures via PCR and Sanger sequencing. By spore genotyping (60 sequences, 10 OTUs), we detected that Sorghum picks up different fungi compared to *Populus* (only 3 overlap OTU between *Populus* root pyrosequencing and Sanger genotyping of *Sorghum* root). Some fungal OTUs were only recovered in *Sorghum* planted with roots of certain *Populus* genotypes. While plant genotypes within a species have little effect on fungal community assemblies, distantly related plants hosts might preferably select for different fungi.
